# Wnt/β-catenin signaling pathway in carcinogenesis and cancer therapy

**DOI:** 10.1186/s13045-024-01563-4

**Published:** 2024-06-18

**Authors:** Pan Song, Zirui Gao, Yige Bao, Li Chen, Yuhe Huang, Yanyan Liu, Qiang Dong, Xiawei Wei

**Affiliations:** 1 Department of Urology, Institute of Urology, West China Hospital of Sichuan University, Chengdu, Sichuan Province 610041 China; 2 Laboratory of Aging Research and Cancer Agent Target, State Key Laboratory of Biotherapy, Cancer Center, West China Hospital, National Clinical Research Center for Geriatrics, Sichuan University, No. 17, Block 3, Southern Renmin Road, Chengdu, Sichuan 610041 P.R. China

## Abstract

The Wnt/β-catenin signaling pathway plays a crucial role in various physiological processes, encompassing development, tissue homeostasis, and cell proliferation. Under normal physiological conditions, the Wnt/β-catenin signaling pathway is meticulously regulated. However, aberrant activation of this pathway and downstream target genes can occur due to mutations in key components of the Wnt/β-catenin pathway, epigenetic modifications, and crosstalk with other signaling pathways. Consequently, these dysregulations contribute significantly to tumor initiation and progression. Therapies targeting the Wnt/β-catenin signaling transduction have exhibited promising prospects and potential for tumor treatment. An increasing number of medications targeting this pathway are continuously being developed and validated. This comprehensive review aims to summarize the latest advances in our understanding of the role played by the Wnt/β-catenin signaling pathway in carcinogenesis and targeted therapy, providing valuable insights into acknowledging current opportunities and challenges associated with targeting this signaling pathway in cancer research and treatment.

## Introduction

The Wnt/β-catenin signaling pathway, also referred to as the canonical Wnt signaling pathway, is a conserved and pivotal axis in orchestrating multiple cell-signaling cascades. It plays an indispensable role in a wide range of physiological processes encompassing proliferation, differentiation, apoptosis, migration, invasion and tissue homeostasis [1–3]. An increasing number of studies have substantiated that the dysfunction of the Wnt/β-catenin pathway contributes significantly to the pathogenesis of various diseases, including solid tumors, hematological malignancies, and sarcoma [4–6]. Biochemical and genetic studies have long elucidated the crucial role of abnormal transcriptional regulation in the early stage of carcinogenesis [6, 7]. With increasing evidence supporting the involvement of the Wnt/β-catenin pathway in carcinogenesis, researchers envision its potential to pave the way for targeted drug development in cancer treatment. Several inhibitors, such as cinobufacini, curcumin, vitamin D, sulindac, and glutaminase 1, have already been identified for impeding carcinogenesis [8–12]. However, the majority of these findings are currently in the preclinical research stage [13]. Therefore, there remains a substantial journey ahead to develop efficacious medications targeting the Wnt/β-catenin signaling pathway. In this review, our objective is to present a comprehensive perspective on Wnt/β-catenin as one of the most crucial mediators in carcinogenesis and provide insights into novel strategies targeting this pathway.

## Overview of the Wnt/β-catenin signaling pathway

The Wnt gene is synonymous with the Drosophila segment polarity gene Wingless and the murine proto-oncogene integration 1, which was molecularly characterized from mouse tumor cells in 1982 [14]. This signaling pathway is a family consisting of two distinct pathways: the noncanonical and canonical pathways. The noncanonical Wnt pathway, which exhibits high diversity, mainly includes the planer cell polarity pathway, Wnt-RAP1 signaling pathway, Wnt-Ror2 signaling pathway, and Wnt-PKA pathway among others. Their crucial role in development has been established by extensive research [15, 16], although further investigations are warranted. In contrast, the canonical Wnt pathway involves β-catenin as the nuclear effector of the transduction pathway.

The Wnt/β-catenin pathway is a highly conserved signaling cascade that spans across species from Drosophila to mammals. Its activation is dependent on the binding of Wnt ligands to frizzled serpentine receptors (FZD), initiating this pathway [17–19]. The Wnt family consists of numerous members with 19 Wnt genes in humans [20]. Wnt proteins play a pivotal role in executing diverse biological functions [21]. FZD protein are seven-span transmembrane receptors that are associated with low-density lipoprotein receptor 5 (LRP5) and LRP6, acting as co-receptors for FZD [17, 19, 22, 23]. Upon binding of Wnt to FZD and LRP5/6 receptors, the signaling pathway is initiated [24]. The downstream effector in this cascade is β-catenin which serves as a vital component of the canonical Wnt pathway [25]. In the absence of Wnt binding, cytoplasmic β-catenin undergoes phosphorylation by a destruction complex (DC) consisting of adenomatous polyposis coli (APC), Axis inhibition protein (AXIN), glycogen synthase kinase 3β (GSK3β), casein kinase 1α (CK1α), and E3 ubiquitin ligase β-TrCP (SCFβ-TrCP) [26]. Within this degradation complex, GSK3β and CK1α facilitate the phosphorylation of β-catenin, resulting in its ubiquitination and subsequent proteasomal degradation [27, 28]. In the presence of Wnt binding, the induction of dishevelled (DVL) leads to complex aggregation at the receptor [29]. Subsequently, phosphorylation and inhibition of GSK3β result in an increase in intracellular β-catenin concentration. Cytoplasmic β-catenin is then transported to the nucleus, where it accumulates and interacts with T cell-specific factor (TCF)/lymphoid enhancer-binding factor (LEF) and co-activators to activate Wnt target genes.

### Components of the Wnt/β-catenin signaling pathway

**Fig. 1:**
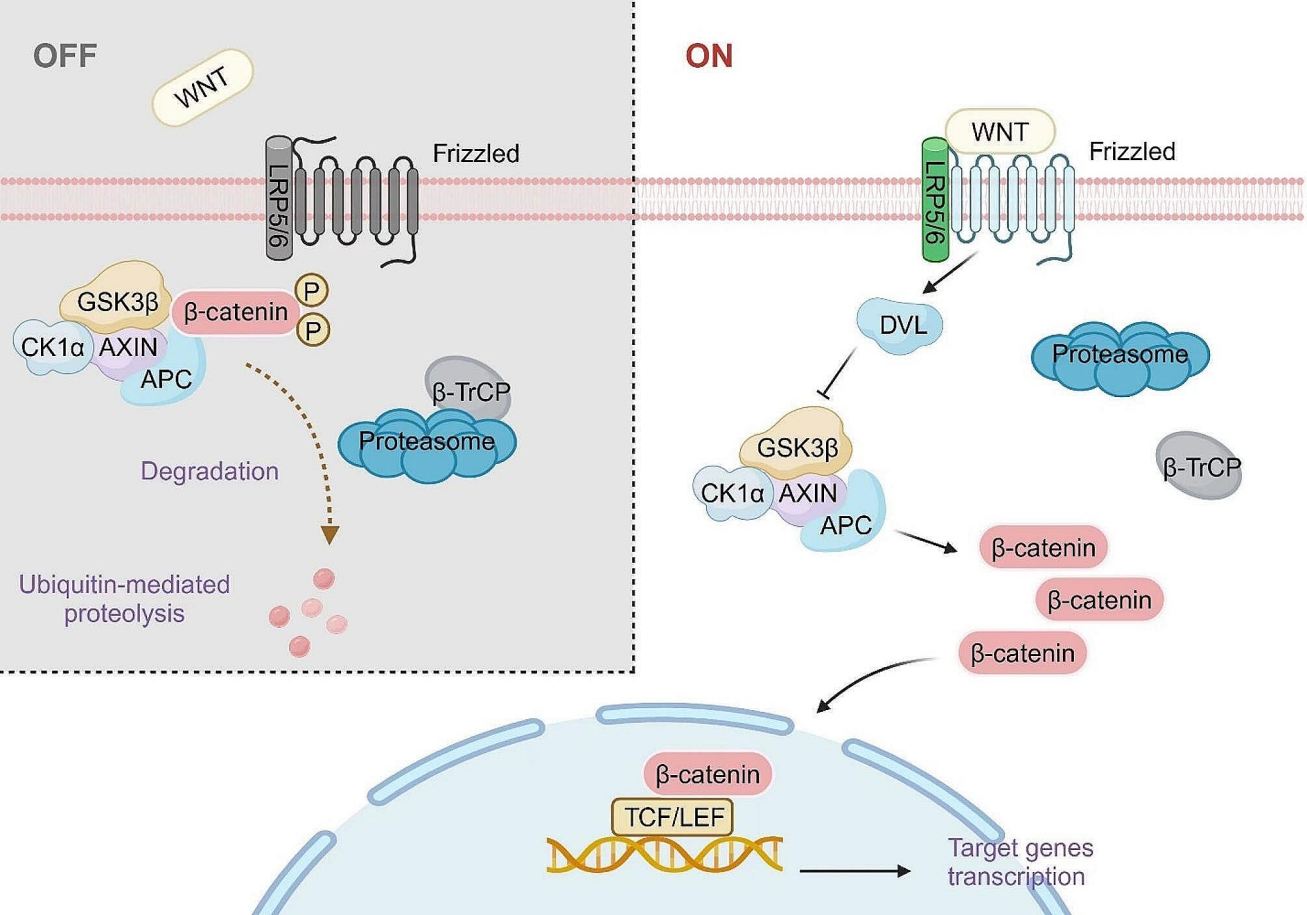
Wnt/β-catenin signaling pathway mechanism. Under normal physiological conditions, cytoplasmic β-catenin is phosphorylated by a destruction complex composed of APC, AXIN, GSK3β, CK1α, and the E3 ubiquitin ligase β-TrCP. In this degradation complex, GSK3β and CK1α mediate the phosphorylation of β-catenin, leading to its ubiquitination and subsequent proteasomal degradation. In the presence of Wnt binding, the FZD receptor is activated, which subsequently recruits DVL to the plasma membrane. DVL then interacts with AXIN, leading to the recruitment and accumulation of a complex at the receptor site. This receptor complex inhibits the activity of the β-catenin destruction complex (DC). As a result, β-catenin accumulates in the cytoplasma and is then transported to the nucleus, where it accumulates and interacts with TCF/LEF and coactivators to activate Wnt target genes

The Wnt/β-catenin pathway consists of four segments: extracellular signaling, membrane segment, cytoplasmic segment, and nuclear segment (Fig. 1). Extracellular signaling is predominantly mediated by Wnt proteins. The membrane segment mainly consists of the Wnt receptor FZD and LRP5/6. The cytoplasmic segment primarily encompasses β-catenin, DVL, GSK3β, AXIN, APC, and CK1. The nuclear segment mainly involves β-catenin translocated into the nucleus, TCF/LEF family members, and downstream target genes. The main components of the Wnt/β-catenin signaling pathway were summarized in Table 1.

#### Extracellular signaling

The Wnt protein family consists of 19 types of secreted proteins found in the human body [30]. The initiation of Wnt/β-catenin signaling primarily relies on Wnt1, Wnt2, Wnt3, Wnt3a, Wnt8b, Wnt10a, Wnt10b, etc. The classical Wnt pathway is typically highly conserved and activated by the secretion/paracrine method, where extracellular Wnt ligands bind to membrane receptors. The conserved cysteines within the structure of Wnts undergo palmitoylation, a process mediated by Porcupine (PORCN) in the endoplasmic reticulum, leading to their modification into lipid-bound forms. The posttranslational modification of Wnts comprises lipid modification and glycosylation. Lipid modification is indispensable for the activity of Wnts [21], whereas glycosylation, particularly N-glycosylation, is not indispensable for Wnt secretion and activity, nor does it possess essentiality for its biological function. Following that, the lipid-modified Wnts are transported from the endoplasmic reticulum to the Golgi apparatus by members of the p24 protein family, including TMED2/CHOp24, TMED4/éclair, and TMED5/opossum. Subsequently, Wnts are transported via an intracellular-dependent manner by Wntless and released into the extracellular matrix via exosomes. Wntless (WLS), also known as Evi and GPR177, is a transmembrane protein encoded by the WLS gene that localizes to the Golgi apparatus. It is essential for the binding of lipid-modified Wnts and facilitates endoplasmic reticulum retrograde trafficking [31].

#### Membrane segment

FZD proteins, which are transmembrane receptors located on the plasma membrane, consist of seven domains [32]. Among these domains, the extracellular cysteine-rich domain (CRD) and the intracellular KTxxxW domain play pivotal roles in FZD function. The CRD is responsible for binding to Wnt ligands, while the KTxxxW domain interacts with the PDZ domains of DVL proteins [33, 34]. LRP5/6 are low-density lipoprotein receptor-related proteins on the plasma membrane, functioning as coreceptors for Wnts [35]. Within the intracellular domain of LRP5/6, the serine/threonine phosphorylation of the PPPSxPS motif is conserved, providing a docking site for AXIN binding [36] as well as presenting a critical step in initiating Wnt/β-catenin signal transduction [37, 38]. When Wnt ligands bind, DVL is recruited to the plasma membrane, contributing to the formation of clathrin-coated pits, thereby facilitating FZD clustering and LRP6 phosphorylation [29, 33, 39, 40].

#### Cytoplasmic and nuclear segment

DVL proteins, residing in the cytoplasm, consist of three critical functional domains. The N-terminal domain is the DIX polymerization domain, responsible for interacting with AXIN. The C-terminal domain is the DEP domain, which functions in the regulation of small GTPases. Additionally, the PDZ binding domain enables interaction with other proteins [34, 41]. When the Wnt/β-catenin signaling pathway is inactive, the formation of the β-catenin DC occurs through the concerted action of AXIN, APC, GSK3β and CK-1α in the cytoplasm. As a multidomain scaffolding protein, AXIN contains binding domains for other DC components, including the DIX domain at the C-terminus and the RGS domain at the N-terminus. The RGS domain specifically interacts with the APC protein. The APC protein also functions as a scaffold in conjunction with AXIN, promoting the phosphorylation of β-catenin by CK1α and GSK3β. CK1, an initiating kinase, initially phosphorylates β-catenin at the Ser45 site, followed by sequential phosphorylation of β-catenin by GSK3. Consequently, the phosphorylated β-catenin is recognized and degraded by the E3 ubiquitin ligase β-TrCP. GSK3 is a serine/threonine kinase that phosphorylates three serine/threonine residues of β-catenin (Ser33, Ser37, and Thr41) [42]. These phosphorylation events drive ubiquitination and proteasomal degradation of β-catenin, resulting in the maintenance of only a minimal amount of β-catenin in unstimulated cells [43]. Notably, the interaction between GSK3 and β-catenin is facilitated by AXIN1 and APC since GSK3 does not directly bind to β-catenin.

β-catenin is a core component of the canonical Wnt signaling pathway. In the absence of Wnt pathway activation, β-catenin undergoes degradation by the DC in the cytoplasm. With the activation of the Wnt/β-catenin signaling pathway, DVL recruits AXIN and GSK3β to the plasma membrane, thereby inhibiting their functions [39, 44]. This process in turn suppresses phosphorylation-mediated degradation as well as promotes the stabilization and accumulation of β-catenin. Subsequently, β-catenin undergoes nuclear translocation and activates Wnt target genes in a positive manner. Owning to the absence of inherent DNA binding ability in β-catenin, its interaction with target genes relies on co-activators along with the recruitment of transcription factors. Coactivators such as B-cell lymphoma 9 (BCL9), Pygopus and CREB-binding protein (CBP)/p300 possess DNA binding domains and can recruit β-catenin to the appropriate gene regulatory regions, allowing for the activation of target genes. Within the context of the Wnt signaling pathway, the TCF/LEF acts as the principal downstream effector and partner of β-catenin [45]. Additionally, β-catenin possesses the ability to interact with various transcription factors, including hypoxia-inducible factor 1α (HIF1α), forkhead box protein O (FOXO), and members of the sex-determining region Y-box (SOX) family [46].

### Biological function of Wnt/β-catenin signaling pathway

The Wnt/β-catenin pathway plays a pivotal role in determining cell fate, promoting cell proliferation and survival, and facilitating cellular differentiation. Furthermore, this pathway critically regulates embryonic development, stem cell maintenance and self-renewal. The dysregulation of the Wnt/β-catenin signaling pathway frequently contributes to the pathogenesis of numerous severe diseases including cancer and non-cancerous diseases. Upon activation, a wide array of target genes, such as cyclin D1, c-Myc, and Axin2, are actived. These genes are involved in regulating the cell proliferation, migration and stem cell properties. Manipulation of β-catenin expression levels both in vitro and in vivo elucidates its role in suppressing autophagosome formation and directly inhibiting p62/SQSTM1, the gene encoding the autophagic adapter protein p62 [47]. Stem cells are characterized by their inherent capacity for self-renewal and differentiation [48]. Cancer stem cells (CSCs) play a crucial role in tumor development, contributing to tumor initiation, expansion, drug resistance, recurrence, and post-treatment metastasis through their ability to self-renew and differentiate. Signals including Wnt/β-catenin, transforming growth factor-β(TGF-β), Hedgehog and Notch play a crucial role in maintaining the self-renewal ability of CSCs. The importance of Wnt/β-catenin signaling in CSCs has gained widespread acknowledgment. Wnt10b, a member of the Wnt ligand gene family, encodes a secreted protein that selectively activates the highly conserved Wnt signaling cascade. Wnt10b plays a pivotal role in regulating the signaling network governing stemness, pluripotency, and cell fate in various organs [49]. The involvement of Wnt signaling in cancer development primarily stems from its ability to promote the sustained proliferation of CSCs in regions that exhibit resistance to anticancer treatments. Moreover, it facilitates the invasion of CSCs into neighboring tissues and their dissemination into the bloodstream. Inhibiting the Wnt/β-catenin signaling pathway offers an effective approach to regulate CSCs.

#### Regulators targeting Wnt

Numerous factors have been identified to regulate the canonical Wnt pathway, and several regulators have been well-established in their influence on signal transduction, including secreted frizzled-related proteins (sFRPs), Wnt inhibitory factor (WIF), glypicans (GPCs), Tiki, and Notum. The regulatory mechanism of sFRPs and WIF in the canonical Wnt pathway involves their distinct domains. sFRPs possess a Netrin-related motif in the C-terminal region, whereas WIF contains a WIF domain in its C-terminal region. These specific domains facilitate the binding of sFRPs and WIF to the Wnt protein [50, 51]. GPCs, a class of evolutionarily conserved heparan sulfate proteoglycans, have been demonstrated to localize on the plasma membrane and exhibit the ability to bind to Wnt proteins for the regulation of their extracellular distribution and signaling activity [52]. GPC3 is believed to possess a CRD domain at its N-terminal end, which interacts with the middle region of Wnt3a. Additionally, GPC3 regulates the Wnt activation according to the concentration dynamically by interacting with FZD through the heparan sulfate chain [53]. Tiki represents a family of Wnt-specific proteases that would directly inhibit Wnt signaling by hydrolyzing the terminal of Wnt3a and triggering the transformation of Wnt3a into oxidized oligomers, thereby depriving its ability to bind receptors [54, 55]. Notum also functions as an enzyme-like inhibitor of Wnt signaling with evidence suggesting that tumor cells suppress normal cells through secretion of notum [56, 57].

#### Regulators targeting FZD and LRP5/6

In addition to targeting Wnts, several regulators possess the ability to directly or indirectly activate FZD and LRP5/6. These include Roof plate-specific spondins (R-spondins; RSPOs), Norrin, and Dickkopf (DKK), all of which are secreted proteins. R-spondins, consisting of four members (RSPO1-4), function as enhancers in the Wnt/β-catenin signaling pathway and are considered indispensable factors in carcinogenesis [58]. This effect was initially discovered in Xenopus embryos, and it was also observed that mouse R-spodin1-3 exhibits a similar effect [59]. R-spondins activate the pathway by binding to FZD and induce the signaling. Furthermore, they can inhibit the activity of Ring finger protein 43 (RNF43) / Zinc and ring finger 3 (ZNRF3) by binding to them, which are ubiquitin ligases located on the plasma membrane responsible for multiubiquitination of lysine [60]. The ubiquitination of FZD resulting from RNF43/Znrf3 leads to FZD endocytosis and subsequent downregulation of Wnt signaling [61–63]. The Norrin protein, which mimics the fingerlike-loop structure of Wnt, exhibits the capability to bind to both the CRD domain of FZD4 and the ectodomain of LRP5/6, forming a ternary complex that facilitates downstream signal transmission of Wnt/β-catenin [51, 64, 65]. The DKK family is widely regarded as an inhibitor of the canonical β-catenin-dependent Wnt pathway [66]. The mechanism by which DKKs inhibit canonical Wnt signaling involves their binding to LRP5/6 and FZD, along with Kremen, a family of two transmembrane proteins characterized by their kringle domains. This three-component complex would contribute to rapid endocytosis and subsequent removal of this protein from the plasma membrane [67, 68]. Therefore, DKKs would turn down the Wnt/β-catenin signaling, offering a negative feedback mechanism to manipulate Wnt signaling [69, 70].

#### Regulators targeting DVL

Two forkhead box (FOX) transcription factors, FOXK1 and FOXK2, have also been discovered as bona-fide DVL-interacting proteins that promote Wnt/β-catenin signaling by translocating DVL into the nucleus [71]. FOXK2, equipped with its forkhead-associated domain (FHA) and adjacent region containing a hydrophobic motif (Leu-137-Phe-145-Phe-154), exhibits the capability to interact with DVL2. This interaction is regulated by DVL phosphorylation, which is contingent upon CK1 and MARK kinases [71]. Disabled-2 (Dab2) functions as an endocytic adaptor protein that is widely expressed. There is substantial evidence supporting its involvement in multiple signaling pathways mediated by receptors [72]. In the context of the Wnt pathway, Dab2 acts as a negative regulator by acting as a scaffold protein that facilitates the degradation of the Wnt receptor complex. Additionally, Dab2 has been suggested to regulate Wnt signaling by interacting with DVL and AXIN, thereby contributing to the regulation of the Wnt/β-catenin signaling pathway.

#### Regulators targeting DC

Dab-2 could bind AXIN with its PTB domain to prevent the dephosphorylation of AXIN resulting from protein phosphatase 1 (PP1), which would contribute to the degradation of β-catenin [73]. PP1 is able to dephosphorylate AXIN, which would lead to the destabilization of AXIN and DC. This process would ultimately result in the accumulation of β-catenin[73]. PP2A is a heterotrimer consisting of a core AC heterodimer combined with a variable regulatory B subunit. Strong evidence supports the notion that the B subunit plays an integral role in the formation of the DC as it exhibits co-immunoprecipitation with AXIN. Moreover, it is epistatically positioned downstream of GSK3β but upstream of β-catenin [74]. The RUNX family, comprising a DNA-binding unit, exerts regulatory effects on the Wnt/β-catenin signaling pathway by either enhancing or reducing its activity. The RUNX family contains a highly conserved domain known as the RUNX domain located at the N-terminus. It is crucial for binding to core binding factor beta (CBFβ) and DNA [75]. The relationship between the RUNX family and Wnts is intricate, as the RUNXs are able to regulate the expression of Wnt, while the β-catenin is able to mediate the expression of RUNX [76]. Twa, a two hybrid-associated protein no.1 with RanBPM, also known as glucose-induced degradation protein 8 homolog, has been identified in colorectal carcinoma tissues [77]. When the Wnt signal is turned off, Twa1 binds to the AXIN complex with β-catenin, resulting in its ubiquitination-mediated degradation. However, upon activation of the Wnt pathway, Twa1 facilitates β-catenin nuclear retention and enhances expression of downstream target genes associated with the Wnt/β-catenin signaling cascade. This effect is mediated by its conserved CRA (CT11-RanBPM) domain, which interacts with β-catenin [78].

#### Regulators targeting β-catenin

ICAT is an 81-amino-acid protein that binds to β-catenin and TCF, which has been identified as an inhibitor of β-catenin signaling because its overexpression effectively hampers the formation of the β-catenin-TCF complex. Furthermore, ICAT acts as a natural inhibitor of APC, thereby preventing APC-mediated degradation of β-catenin and exerting a positive effect on Wnt signaling [79]. Kdm2a/b are protein lysine demethylases located in the nucleus. With their fourth and fifth armadillo repeats, they possess the ability to modulate the methylation/demethylation of nuclear β-catenin, thereby regulating Wnt/β-catenin signaling [80]. The impact of Kdm2a on this process is concentration-dependent, as an elevated level of Kdm2a significantly enhances the ubiquitylation of β-catenin. Upon binding of Wnt to FZD, non-phosphorylated β-catenin translocates to the nucleus followed by methylation at lysine residues within the fourth and fifth armadillo repeats. Subsequently, the modified β-catenin forms a complex with the TCF/LEF transcription factor to activate transcription of the target gene. In this context, Kdm2a/b competes with TCF/LEF for β-catenin binding and elicits the degradation of β-catenin. Importantly, evidence suggests that even without demethylation, Kdm2a/b-mediated destruction of the β-catenin/TCF complex inhibits Wnt signaling [80].

**Table 1:** The main components of the Wnt/β-catenin signaling pathway

Components	Full name	Role in Wnt/β-catenin signaling pathway
Wnt	Wingless and INT1	Main ligands that activate the Wnt/β-catenin signaling pathway
PORCN	Porcupine	A membrane bound O-acyltransferases that adds palmitoleate groups to Wnt proteins
WLS	Wntless	Binding to lipid-modified Wnts and contribute to Wnt secretory
FZD	Frizzled	Wnt ligands bind to Wnt proteins and activate the Wnt/β-catenin signaling pathway
LRP5/6	Lipoprotein receptor-related proteins 5 and 6	Form cell surface receptor complexes with FZD
DVL	Dishevelled	Protein recruited to the FZD–LRP5 and FZD–LRP6 complex in the presence of Wnt ligands and enabling the transduction of Wnt signalling
APC	Adenomatous polyposis coli	Forming the β-catenin destruction complex with CK1, AXIN, GSK3β
AXIN	Axin	Forming the β-catenin destruction complex with CK1, APC, GSK3β
CK1α	Casein kinase 1α	Forming the β-catenin destruction complex and phosphorylating the β-catenin at Ser45
GSK3β	Glycogen synthase kinase 3β	Forming the β-catenin destruction complex and phosphorylating the β-catenin at Thr41, Ser33, Ser37
β-catenin	β-catenin	A central molecule in the Wnt/β-catenin signaling pathway that translocates from the cytoplasm to the nucleus and mediates TCF–LEF-dependent gene expression
TCF/LEF	T cell factor–lymphoid enhancer factor	Transcriptional factors that bound to β-catenin in the nucleus and activate target gene transcription.
β-TrCP	β-transducin repeat-containing protein	Binding to phosphorylated β-catenin and leading to its degradation
WIF	Wnt inhibitory factor	Binding with Wnt proteins and preventing its interaction with the receptor complex
sFRP	Soluble frizzled-related protein	Extracellular inhibitors of Wnt signaling by binding to Wnt ligands and preventing their interaction with Frizzled receptors
GPCs	Glypicans	Co-receptors for Wnt ligands by interacting with both the Wnt ligands and the Frizzled receptors
Tiki	Tiki	A membrane bound proteases that cleave and inactivate Wnt proteins
Notum	Notum	Inhibiting the activation and secretion of Wnt ligands by removing the lipid modification of palmitoleate in Wnt proteins
R-spondin	Roof plate-specific spondins	The ligands for LGR4/5 and upregulation of Wnt signalling
Norrin	Norrin	A ligand interacts with FZD4 and LRP5/6 leading to the activation of downstream signaling
RNF43	Ring Finger Protein 43	A negative regulator of the Wnt pathway by promoting the degradation of the Frizzled/LRP5/6 complex
DKKs	Dickkopf	Antagonists of the Wnt signaling pathway by inhibiting the formation of the Wnt-Frizzled-LRP5/6 complex.
Dab2	Disabled-2	Negative regulator of the Wnt signaling by facilitating the degradation of the Wnt receptor complex and interacting with DVL and AXIN
FOXK1/ FOXK2	Forkhead box protein K1/K2	Transcriptional co-repressors of Wnt target genes and DVL-interacting proteins by translocating DVL into the nucleus
PP2A	Protein phosphatase 2 A	A protein phosphatase that dephosphorylates β-catenin

## Wnt/β-catenin signaling pathway and carcinogenesis

**Fig. 2:**
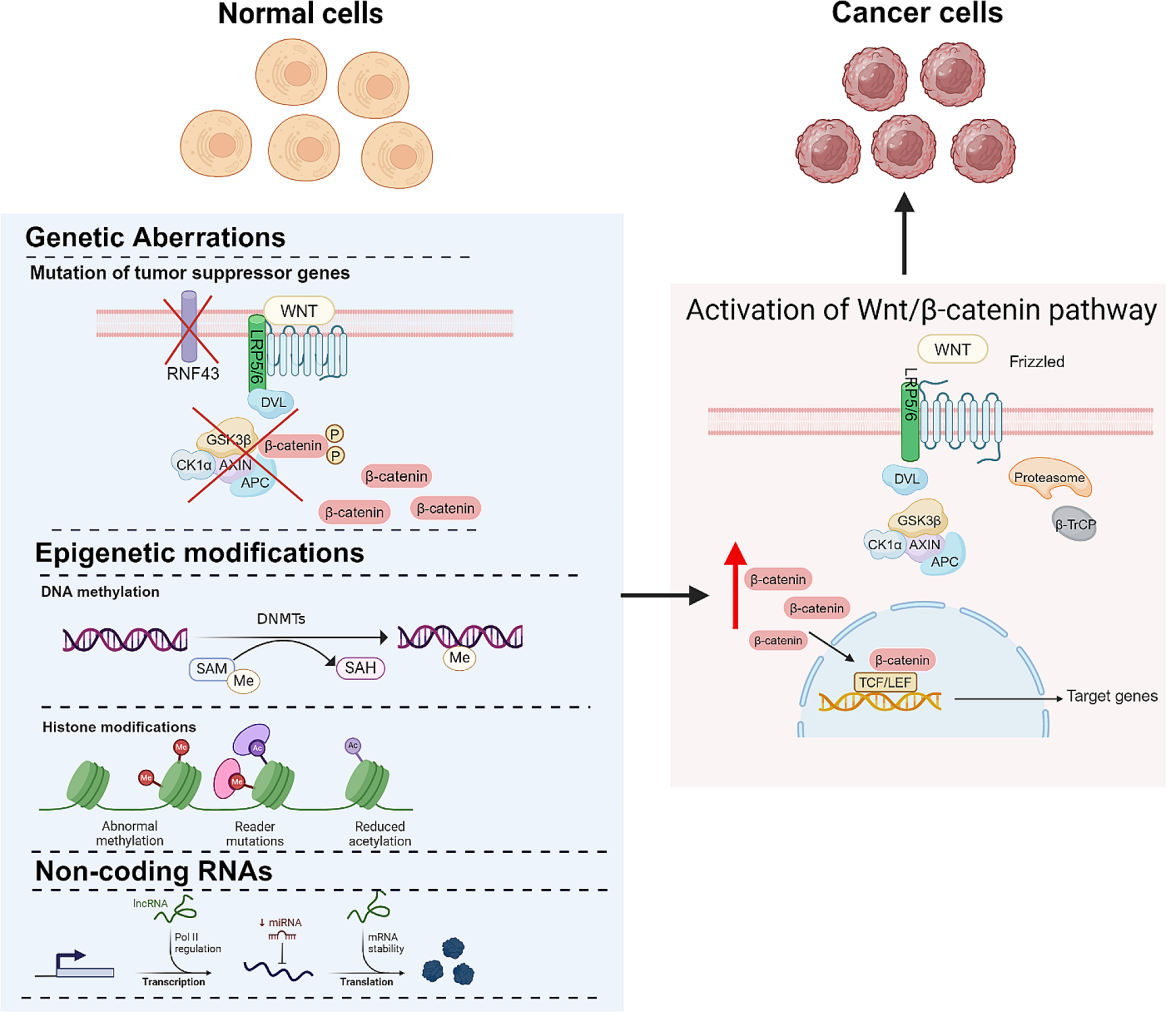
Aberrant Wnt/β-catenin signaling pathway activation in tumor. Genetic abnormalities and epigenetic modifications can activate the Wnt/β-catenin pathway, leading to the onset of tumorigenesis. Mutations of tumor suppressor genes decrease the activity of the destruction complex, contributing to tumor growth. RNF43 loss-of-function mutations that increase Wnt receptor abundance on the cell surface, rendering tumors sensitive to Wnt inhibitors and decreasing negative feedback at the receptor-level to drive tumor growth. Loss of DNA methylation results in aberrant transcription of target genes, including WIF1, SFRP, DKK1, DACT, SOX7/17, β-TrCP, E-Cadherin, APC, AXIN-2, Wnt7a/9A. Histone modifications, such as methylation, acetylation, and phosphorylation, regulate the Wnt/β-catenin signaling pathway in tumors by controlling chromatin accessibility and gene expression of Wnt pathway components and downstream targets, impacting tumor initiation, progression, and metastasis. Non-coding RNA, including lncRNA and miRNA, modulate the Wnt/β-catenin signaling pathway in tumors by targeting key components such as β-catenin, Wnt ligands, receptors, and genes

Genetic and epigenetic alterations of the Wnt/β-catenin pathway can induce aberrant activation, ultimately contributing to the development of cancer. The persistent activation of the Wnt/β-catenin pathway in cancer is associated with various oncogenic processes including increased cell proliferation and epithelial-mesenchymal transition (EMT), as well as the maintenance of self-renewal capacity of CSCs. The identification of APC mutations in colon cancer pathogenesis back in 1991 provided the initial evidence for the involvement of Wnt/β-catenin signaling in tumorigenesis. Dysregulated Wnt signaling has been increasingly demonstrated to be associated with a range of cancers including liver, lung, and breast cancers [81]. Multiple mechanisms are responsible for driving the activation of the Wnt/β-catenin pathway, encompassing mutations in key pathway components, deregulation of Wnt ligands and receptors, and epigenetic modifications such as DNA methylation and histone modifications (Fig. 2).

### Mechanisms of activation of the Wnt/β-catenin signaling pathway in tumors

#### Wnt tumor suppressor mutations

Inactivation mutations of the component comprising DC represent a prevalent method of carcinogenesis. The APC gene, initially identified as a mutated gene in familial adenomatous polyposis coli (FAP), is also detected in 80% of colorectal adenomas and colorectal carcinomas (CRCs), positioning it as one of the earliest mutations in the development of colon cancer [82, 83]. Mutations in the APC gene do not result in a complete loss of protein but rather result in the production of truncated proteins owning to premature termination codons. Consequently, this results in the activation of the Wnt signaling pathway [83]. Additionally, these mutations disrupt various cellular processes, including loss of intercellular adhesion [84], impaired DNA repair within the nucleus, chromosomal destabilization during mitosis [83], and compromised anti-apoptotic functions through transcriptionally non-dependent mechanisms [85]. AXIN has been identified with mutations in various carcinoma types including hepatocellular carcinoma (HCC), CRC, medulloblastomas, and ovarian endometrioid adenocarcinoma [86–91]. Recent studies have demonstrated that adenovirus-mediated transfer of wild-type AXIN1 gene can elicit apoptosis in HCC and CRC cells that have accumulated β-catenin due to APC, CTNNB1 or AXIN1 mutation [89]. RSK2-inactivating mutations frequently co-occur with AXIN1-inactivating or β-catenin-activating mutations, which play a synergistic role in promoting HCC development [92].

RNF43, as a E3 ubiquitin-protein ligase, exerts inhibitory effect on Wnt/β-catenin by mediating FZD ubiquitination followed by lysosomal degradation. Its mutation is frequently observed in several carcinoma types including HCC, pancreatic adenocarcinoma, and CRC [93–95]. The absence of RNF43 can enhance resistance to γ-radiation and chemotherapy of gastrointestinal cancers cells by inhibiting the activation of DNA damage response [96]. In pancreatic adenocarcinoma, the RNF43 F69C mutation is associated with a significantly reduced expression of FZD compared to wild-type cells, highlighting the importance of RNF43 in pancreatic adenocarcinoma development [97]. An analysis investigating inactivating mutations of RNF43 that confer Wnt dependency in pancreatic ductal adenocarcinoma suggests that BRAF, ARID1A, RNF43, and KM2B mutations exhibit the highest frequency in microsatellite instability (MSI) colon cancer. These gene mutations are considered as pivotal gene mutations associated with the microsatellite status in colon cancer [98]. The mutation of CTNNB1 which is responsible for encoding β-catenin, is associated with nuclear localization and activation of Wnt/β-catenin signaling. Hotspot mutations in Exon 3 of CTNNB1 alter the N-terminus of β-catenin, impeding its phosphorylation and degradation by DC, thereby resulting in β-catenin accumulation [99]. A meta-analysis identified that with CTNNB1 S45F mutation, the desmoid tumor is more likely to reoccur [100]. In parathyroid adenomas, DNA sequencing demonstrates the presence of stabilizing homozygous mutations in 7.3% of patients while aberrant β-catenin accumulation is commonly observed at a high frequency [101]. Furthermore, it has been elucidated that the expression of CTNNB1 mutation is associated with poorer overall survival in low-grade endometrioid endometrial carcinoma [102].

#### Epigenetic modifications

Cells exhibit genetic uniformity, yet manifest phenotypic variability in terms of structure and function. These heritable modifications, which do not involve alterations to the DNA sequence, are designated as epigenetic changes. Epigenetic modifications respond to a wide spectrum of signaling cues, encompassing DNA methylation, histone variants, histone modifications, chromatin structure, nucleosome remodeling, and interactions among various epigenetic factors (Table 2).

**Table 2:** Epigenetic modifications regulating the Wnt components

Wnt component	Epigenetic alteration	Cancer type	Outcome	Ref.
Wnt2	Loss of H3K27me3	Colorectal cancer	Upregulation	[103]
Wnt5a	Promoter hypermethylation	Colorectal cancer	Downregulation	[104]
W10b	Promoter hypermethylation	Colorectal cancer	Downregulation	[105]
DKK1	Promoter hypermethylation	Colorectal cancer, Breast cancer	Downregulation	[106–109]
DKK3	loss of H3Ac loss of H3K4me3	Glioma, Breast cancer Colorectal cancer	Downregulation	[106–108]
sFRP1	Promoter hypermethylation	Colorectal cancer, Breast cancer	Downregulation	[110–113]
sFRP2	Promoter hypermethylation	Colorectal cancer, Hepatocellular carcinoma, Breast cancer	Downregulation	[114, 115]
sFRP3	Promoter hypermethylation	lung adenocarcinoma	Downregulation	[116]
WIF1	Promoter hypermethylation	Colorectal cancer, Breast cancer, Endometrial carcinogenesis	Downregulation	[117–120]
FZD4	Histone H3K9 acetylation	Colon cancer	Upregulation	[121]
FZD10	Gain of H3K9Ac	Breast cancer	Upregulation	[103]
APC	Promoter hypermethylation	Colorectal cancer	Downregulation	[122, 123]
AXIN2	Promoter hypermethylation	Colorectal cancer	Downregulation	[104, 124, 125]
β-catenin	Lysine acetylation	Colorectal cancer Breast cancer	Upregulation	[126, 127] [128, 129]
LRP6	Lysine acetylation	Colorectal cancer	Upregulation	[130, 131]
TCF4	Lysine acetylation	Colorectal cancer	Upregulation	[132]
GSK3β	Lysine acetylation	Neuroblastoma	Upregulation	[114, 115]

#### DNA methylation

DNA methylation has been demonstrated to be an essential epigenetic modification regulating gene expression, thereby implicating its involvement in numerous cellular processes. DNA methylation exerts its influence on multiple regulators within the Wnt/β-catenin pathway. DNMT1 is a key mediator for the proper functioning of β-catenin, which is responsible for the formation of the DNMT1-β-catenin complex to mediate DNMT-dependent promoter methylation and targeted gene expression within the Wnt/β-catenin pathway [133]. In HCC, evidence suggests that the expression of BEX1, a human oncofetal protein as well as a stem-cell marker in liver cancer, is capable of interacting with RUNX3 to block its inhibitory effect on β-catenin. More importantly, the variation of expression BEX1 in different types of HCC has been certified to be associated with DNMT1-mediated DNA methylation [134]. Na^+^/H^+^ exchanger regulatory factor 1, an adaptor molecule known to suppress Wnt signaling has been identified to be downregulated in CRC cells due to the DNMT1-mediated promotor methylation [135]. Additionally, the significant effect of DNA methylation on the components of the Wnt/β-catenin pathway has been discovered. In recurrent glioblastoma (GBM) patients, the methylation levels of the promoters and genes of Wnt5a, β-catenin and Wnt3a are lower in comparison with the primary GBM patients. Conversely, FZD-10 exhibits a uniform high methylation level. Notably, hypermethylation of the Wnt5a promoter is universally observed in CRC tissues [103, 104]. The methylation of DKK is also frequently observed in cancer tissues including gastric carcinoma, HCC and cervical squamous cell carcinoma. It has been proven to be related to poor prognosis [106–108]. DC is frequently subjected to methylation, representing a commonly observed phenomenon. In lung carcinoma, APC promoter methylation has been correlated with smoking status and non-metastatic cases [122], while methylation in APC has been observed to be associated with an increased risk of prostate cancer-specific mortality [123]. As a crucial component of DC, methylation of AXIN2 leads to the silencing of AXIN2 expression, particularly in MSI CRC specimens [124].

#### Lysine acetylation

Lysine acetylation is a crucial protein modification that enables the reversible regulation of target protein function, contingent upon the activity of lysine acetyltransferases (KATs) and the catalytic function of lysine deacetylases (KDACs). A growing body of evidence suggests that dysregulation of lysine acetylation and subsequent activation of the Wnt/β-catenin pathway contribute to cancer development and progression. Notably, various cancers exhibit overexpression of KATs alongside the downregulation of KDACs, creating an advantageous environment for lysine acetylation-mediated Wnt/β-catenin pathway activation. This process can be categorized into histone acetylation and non-histone acetylation, both playing a critical role in the activation of the Wnt/β-catenin signaling pathway. Furthermore, studies have demonstrated that targeting lysine acetylation holds promise in suppressing tumor growth and sensitizing cancer cells to chemotherapy, highlighting its potential as a therapeutic strategy.

In the Wnt/β-catenin signaling pathway, the acetylation of four specific molecules, namely LRP6, TCF4, GSK3β, and β-catenin, has been elucidated. The acetylation of LRP6 is promoted by p300, subsequently triggering its phosphorylation for signal responsiveness [131]. TCF4 undergoes acetylation at K150 in conjunction with CBP, leading to conformational changes in the TCF4-DNA complex [132]. GSK3β, a component of DC involved in this signaling pathway, has also been identified to undergo acetylation. Studies have demonstrated that the members of sirtuin (SIRT) family of deacetylases including SIRT1, SIRT2, and SIRT3 can mediate deacetylation to inhibit GSK3β activity [114, 115]. It has been discovered that the acetylation of β-catenin is associated with CBP, p300, and PCAF. Specifically, the acetylation of β-catenin at K345 is linked to the involvement of p300 [127], while the acetylation of K49 is correlated with CBP [128]. Additionally, both K19 and K49 play crucial roles as essential residues during the acetylation process mediated by PCAF [129]. Notably, β-catenin acetylation not only enhances its stability by inhibiting ubiquitin-mediated degradation but also promotes its nuclear translocation, strengthens its interaction with TCF, and further augments transcriptional activation of Wnt-dependent genes. The interaction between SIRT6 and FZD4 leads to the suppression of FZD4 transcription by reducing histone H3K9 acetylation in HCC [121]. Similarly, in breast cancer, the overexpression of prostate tumor overexpressed-1 has been found to inhibit DKK1 transcription through the recruitment of histone deacetylase 1(HDAC1) and HDAC2, resulting in decreased levels of histone H3/H4 acetylation at the DKK1 promoter [109]. Nonetheless, the regulatory effects of acetylation on other molecules within the DC remain incompletely elucidated [136].

#### Noncoding RNAs that regulate Wnt/β-catenin signaling pathway

MicroRNAs (miRNA) are a class of noncoding endogenous small RNAs, consisting of 22 nucleotide sequences. Evidence suggests that several miRNAs have the ability to activate the Wnt/β-catenin signaling pathway by inhibiting key components as shown in Table 3. For instance, miR-135b has been identified as capable of activating β-catenin expression in osteosarcoma cells and enhancing the Wnt/β-catenin signaling [137]. Similarly, miR-388-5p has been verified to downregulate the expression of WIF1 while promoting Wnt8 expression, thereby leading to subsequent activation of this pathway in pancreatic cancer [138]. In breast cancer, miR-183 has been identified as a direct inhibitor of key components of the DC, including AXIN1, AXIN2, and GSK3β, to activate the canonical Wnt signaling [139, 140]. The overexpression of miR-374a in breast cancer enhances the nuclear translocation of β-catenin, thereby promoting the transcriptional activity of TCF/LEF [141]. Evidence suggests that miR-106a-3p, miR-494, miR-100, miR-125b, and miR-155 possess the capacity to directly inhibit APC, leading to β-catenin translocation and subsequent transcriptional activation of target genes [142–145]. In addition, miR-455-3p and miR-1246 have been identified to target GSK3β [103,104], while miR-128-3p has been certified to regulate AXIN [146]. Evidence demonstrated that miR-410 possesses the ability to effectively inhibit DKK1 and DKK3, thereby facilitating the translocation of β-catenin into the nucleus [147]. MiR-128-3p plays an important role in promoting the metastasis of non-small cell lung cancer (NSCLC) by downregulating the expression of AXIN1, SFRP2, and WIF1 [148]. MiR-182-5p functions as an inhibitor of FOXO3a expression by impeding the interaction between FOXO3a and β-catenin. This inhibition subsequently potentiates the interaction between β-catenin and TCF4, ultimately leading to Wnt signaling activation [149]. In endometrial cancer, the expression of RORA is downregulated by miR-652 followed by the activation of Wnt/β-catenin signalling [150]. In CRC, RAS association domain family 6 (RASSF6) is evidenced to be inhibited by miR-496, resulting in the Wnt/β -catenin signaling activation along with enhanced migration and EMT of CRC [151].

Long-chain non-coding RNAs (LncRNAs) represent the most extensively distributed and heterogeneous class of non-coding RNA molecules, playing a crucial role in regulating the Wnt/β-catenin pathway (Table 3). The lncRNA-CRNDE has been observed to be overexpressed in various cancer cells. It is capable of promoting EMT of HCC cells through the upregulation of Wnt2, FZD4 and β-catenin expression [152–154]. Given the significant role of DC in the Wnt/β-catenin pathway due to their complex structure with multiple vulnerable sites, lncRNA-JPX has been utilized to impair the function of DC by inhibiting the expression of GSK3β. lncRNA-LALR1 exhibits the ability to attenuate the level of AXIN1 in hepatocytes by recruiting CTCF, a transcription factor in chromatin organization and gene regulation. Consequently, this event leads to an elevation in cytoplasmic β-catenin concentration, subsequently enhancing the transcription of c-Myc and Cyclin D1 [155]. A number of lncRNA have been identified to foster Wnt/β-catenin signaling by modulating its regulators. An example in point is AP-2α, a negative regulator that impedes the interaction of β-catenin and TCF4. lncRNA-CCAL has been verified to target AP-2α, giving rise to MDR1/P-gp expression upregulation as well as the development of multi-drug resistance (MDR) in CRC [156]. DDX5 functions as a positive regulator for β-catenin, facilitating its expression and safeguarding it against degradation. In various cancer categories, it has been observed that lncRNA-NEAT1 is able to interact with DDX and augment gene transcription [157]. LncRNA-β-Catm presents the capacity for association with methyltransferase EZH2 followed by an increase in β-catenin methylation. This event improves β-catenin stability and promotes Wnt/β-catenin signaling [158]. In addition, certain lncRNAs are able to interact with microRNA and further regulate the pathway. For example, it has been validated that lncRNA-LINC01133 inhibits miR-106a-3p in gastric cancer, thereby regulating the Wnt signaling pathway negatively [144]. lncRNA-CRNDE has also been observed to play its role in inhibiting miR-181a-5p, thereby promoting the progression and chemoresistance of CRC [159]. It has been reported that lncRNA-LINC01606 can compete with miR-423‐5p, contributing to the upregulation of stearoyl‐CoA desaturase 1 and activation of the canonical Wnt/β‐catenin signaling pathway [160].

**Table 3:** Noncoding RNAs that regulate Wnt/β-catenin signaling pathway

ncRNAs	Regulated molecules	Related tumors	Ref.
miR-135b	β-catenin	Ovarian cancer	[137]
miR-388-5p	WIF1, Wnt8	Pancreatic cancer	[138]
miR-31	DKK1	Breast cancer	[139]
miR-183	AXIN2	Breast cancer	[140]
miR-374a	TCF/LEF, WIF1, PTEN, Wnt5a	Breast cancer	[141]
miR-106a-3p	APC	Hepatocellular carcinoma	[142]
miR-494	APC	Colorectal cancer	[143]
miR-106a-3p	APC	Gastric cancer	[144]
miR-100	APC	Colorectal cancer	[145]
miR-125b	APC	Colorectal cancer	[145]
miR-455-3p	GSK3β	Esophageal squamous cell carcinoma	[161]
miR-1246	GSK3β	Hepatocellular carcinoma	[162]
miR-128-3p	AXIN1, SFRP2, WIF1	Non-small cell lung cancer	[163]
miR-410	DKK1, DKK3	Colorectal cancer	[147]
miR-22-3p	β-catenin	Glioblastoma	[164]
miR-22-5p	β-catenin	Glioblastoma	[164]
miR-181a	STAB1	Ovarian cancer	[165]
miR-448	SFRP4	LC	[166]
MiR-145	SOX2	Glioblastoma	[167]
miR200c	DACH1	PTC	[168]
miR-19b	FBXW7	Colorectal cancer	[169]
miR-27a	FOXO1	Ovarian cancer	[170]
miR-128-3p	AXIN1, SFRP2, and WIF1	Non-small cell lung cancer	[148]
miR-182-5p	FOXO3a and β-catenin	Hepatocellular carcinoma	[149]
miR-652	RORA	Endometrial cancer	[150]
miR-496	RASSF6	Colorectal cancer	[151]
lncRNA-CRNDE	Wnt2, FZD4 and β-catenin	Hepatocellular carcinoma	[152–154]
lncRNA-JPX	GSK3β	LC	[171]
lncRNA-LALR1	AXIN	Hepatocellular carcinoma	[155]
lncRNA-CCAL	AP-2α	Colorectal cancer	[156]
lncRNA-NEAT1	DDX5	Colorectal cancer	[157]
lncRNA-β-Catm	EZH2	Colorectal cancer	[158]
lncRNA-LINC01606	TFE3	Colon cancer	[160]

#### Cross-talk between the Wnt/β-catenin pathway and other signaling pathways

##### TGF-β and Wnt/β-catenin pathway

The TGF-β pathway plays a crucial role in regulating cell proliferation, differentiation, and fibrosis tumor genesis. There exist multiple intersecting points between the TGF-β pathway and the canonical Wnt pathway, involving Smad, AXIN, DVL, and β-catenin. TGF-β has the ability to activate the TGF-β/Smad pathway by upregulating Wnt2, Wnt4, Wnt5a, Wnt7a, and Wnt10a as well as the co-receptor LRP5. Additionally, it enhances nuclear accumulation and stability of β-catenin in human marrow stromal cells [172]. Smad can interact with LEF1/TCF transcription factor which is essential for synergistic activation by both TGF-β and the Wnt/β-catenin pathway [173]. In prostate carcinoma, the activity of Stat3 located on the Wnt3a promoter is inhibited by TGF-β, resulting in a reduction of Wnt3a levels in tissues and subsequent suppression of tumor progression [174].

##### Notch pathway and Wnt/β-catenin pathway

The Notch signaling pathway is a conserved ligand-receptor signaling cascade in mammals, encompassing 5 ligands and 4 receptors that exhibit a similar structural framework [175]. Notably, the interaction between Notch and the canonical Wnt pathway occurs through various mechanisms, such as the upregulation of Notch ligand and receptor expression mediated by Wnt signaling. In adult epidermal cells, activation of the Wnt signaling pathway leads to the accumulation of β-catenin, thus initiating Jag1 transcription and subsequently activating Notch signaling. This intricate interplay ultimately leads to the expansion of the base of the hair follicle, sebaceous gland enlargement and abnormal clumping of the follicles [176]. In chronic lymphocytic leukemia (CLL), Notch2 has been regarded as the pre-requisition for the activation of canonical Wnt signaling in tumor cells. This is attributed to the ability of Notch2 to induce C1q transcription in mesenchymal stromal cells, which subsequently inhibits GSK3β-mediated degradation of β-Catenin [177]. Besides, stromal Notch2 activity plays a regulatory role in N-cadherin expression within CLL cells, which interacts with and further stabilizes β-catenin [177]. In contrast, the Notch signal can also exert negative regulation on β-Catenin. In stem and progenitor cells, membrane-bound Notch associates with unphosphorylated β-Catenin, and negatively regulates post-translational accumulation of active β-Catenin protein.

##### Hippo pathway and Wnt/β-catenin pathway

The Hippo pathway consists of a cascade of kinases that govern the phosphorylation of the co-activators of Yes-associated protein (YAP) and a transcriptional coactivator with PDZ-biding motif (TAZ) [178]. The core effectors YAP/TAZ in the Hippo signaling pathway have direct interactions with β-catenin in the Wnt signaling pathway to promote gastrointestinal tumor development [179]. Upon dephosphorylation, YAP and TAZ translocate to the nucleus where they bind to TEA domain transcription factors, thereby activating target genes. Additionally, they function as integral components of the DC and exhibit close interactions with AXIN1, β-TrCP, β-catenin and GSK3β. In the absence of Wnt ligands, TAZ and YAP are recruited alongside β-TrCP, which initiates the degradation of β-catenin [180]. TAZ possesses the ability to inhibit Wnt-induced activity while concurrently inducing the expression of target genes that are dependent on the presence of Wnt3a. Suppression of TAZ results in the stabilization of β-catenin, leading to the accumulation of nuclear β-catenin and phosphorylation of DVL [181]. As a transcriptional co-activator within the Hippo signaling pathway, YAP negatively regulates the Wnt/β-catenin pathway by directly binding to β-catenin and facilitating its effective degradation.

##### Hedgehog pathway and Wnt/β-catenin pathway

The interaction between Hedgehog (HH) and Wnt signaling pathways is believed to be mediated by the endogenous sFRP-1 protein. It has been demonstrated that sFRP-1, induced by HH transcription, functions as a negative regulator of the Wnt pathway [182]. Activation of HH signaling leads to the induction of the expression of TCF7L2 (and human TCF4) isoforms, including dominant negative isoforms, which limit β-catenin signaling. Consequently, this inhibition leads to decreased expression of fibroblast growth factor 18 (FGF18) and the formation of ectopic cartilage. In adults, sustained HH signaling activity results in cartilage degeneration, whereas increasing β-catenin activity may counteract this effect by rebalancing HH and Wnt/β-catenin signaling [183].

##### NF-κB pathway and Wnt/β-catenin pathway

Several studies have reported the negative regulation of the Wnt/β-catenin pathway by NF-κB pathway. Activation of NF-κB has been demonstrated to inhibit the nuclear translocation of β-catenin [184], as well as suppress the activity or expression levels of transcription (co-)factors in the Wnt pathway other than β-catenin [185]. Notably, NF-κB indirectly governs the Wnt/β-catenin pathway by influencing target genes that impact β-catenin’s activity or stability. In colon cancer, liver cancer, and breast cancer cells, NF-κB activation hampers β-catenin/TCF activity through upregulation of leucine zipper tumor suppressor 2, which is conversely downregulated in GBM cells to promote β-catenin/TCF activity [186]. Furthermore, NF-κB promotes the degradation of β-catenin through the induction of E3 ubiquitin ligases SMAD ubiquitin regulatory factor 1 (Smurf1) and Smurf2 [187].

Components of the NF-κB signaling pathway, such as IKK and RelA, have been implicated in the positive regulation of Wnt/β-catenin signaling. Both IKKα and IKKβ, crucial activators of the NF-κB pathway, exhibit distinct modes of regulating β-catenin-dependent transcriptional activity [188]. Research has suggested that IKKα specifically enhances the transcriptional activity of β-catenin/TCF and induces upregulation of CCND1, a downstream target gene encoding cyclin D1 [189]. Moreover, IKKα promotes cytoplasmic stabilization of β-catenin by simultaneously suppressing the GSK3β/APC-mediated canonical degradation pathway and the non-canonical degradation pathway that involves seven in absentia homolog 1 [190].

##### Ras/Raf/Mek/Erk pathway and Wnt/β-catenin pathway

There are three major subtypes of Ras, namely H-Ras, K-Ras, and N-Ras. The activity of these subtypes is regulated by a guanosine diphosphate/guanosine triphosphate loading switch. Both the Ras/MAPK pathway and Wnt/β-catenin pathway have been observed to play crucial roles in tumor development. Evidence indicates that GSK3β mediates the phosphorylation of H-Ras, which is inhibited by Wnt3 but facilitated by AXIN and APC. Phosphorylated H-Ras recruits the E3 ligase linker protein β-trcp for its polyubiquitylation and subsequent degradation [191]. 

### Wnt/β-catenin signaling pathway in carcinogenesis of various tumors

The up-regulation of the Wnt/β-catenin signaling pathway has been implicated in carcinogenesis across various tissues, including colorectal, gastric, and prostate, etc [192]. The Wnt/β-catenin signaling pathway exerts its influence on tumor cell proliferation and apoptotic processes by modulating downstream target genes. Additionally, this pathway is closely connected with the degradation of extracellular matrix, cancer cell migration and adhesion, as well as tumor angiogenesis [193]. Activation of this pathway leads to an increase in the transcription factor SNAI1 levels while suppressing E-cadherin expression through inhibition of GSK3β-mediated β-catenin phosphorylation. Consequently, the induction of EMT promotes tumor metastasis [194]. In addition, the abnormal activation of the Wnt/β-catenin signaling pathway has been shown to facilitate the proliferation, renewal and differentiation of CSCs. This event plays a significant role in the process of carcinogenesis, leading to resistance development to chemotherapy and immune evasion potentially [195].

#### Regulations in specific tumors

##### Colorectal cancer

The Wnt signaling pathway alterations are universally observed in CRC tissues, with more than 90% of CRC cases harboring mutations in genes such as APC, CTNNB1, RNF42, AXIN1, or RSPO [196]. Clinical data indicates that approximately 70% of both sporadic and hereditary colorectal cancers exhibit deletions or mutations in the APC gene [197]. In a cohort of 720 colorectal cancer patients, the deletion of membrane β-cyclins was predominantly associated with a poor prognosis, as evidenced by overall survival analysis [198]. Mutations in the AXIN1 or AXIN2 genes have been detected across various types of cancers [199]. The occurrence of APC mutations has been linked to the initiation of CRC development, as observed in FAP. The APC multiple intestinal neoplasias mouse model has been employed to investigate intestinal cancer [200, 201].

RSPO fusion also plays a crucial role in CRC genesis. The RSPO family comprises four members (RSPO1-4) that share a similar structure enabling interaction with LGR4-6, LPR5/6, ZNRF3/RNF43, FZD, and heparan sulfate proteoglycans [202]. RSPO mutations, gene rearrangements, fusions, copy number alterations, and altered gene expression have also been identified in a variety of cancers including CRC [203]. The PTPRK-RSPO3 fusion transcription is the predominant RSPO type detected in traditional serrated adenoma (TSA), a rare type of colorectal serrated polyp. EIF3E-RSPO2 fusion was identified in CRC and PIEZ01-RSPO2 was also identified in cDNA ends [204]. Hyperactivation of the Wnt pathway sustains proliferative signals and prevents proper differentiation of intestinal stem cells into mature cells, resulting in the accumulation of undifferentiated cells that can contribute to tumor initiation and growth. Several characteristics have been identified in stem cells, including Lgr5, Bmi1, Lrig1 and Dclk1 [205–207]. The ablation of cells expressing Bmi1 or directly elimination of Lgr5-positive cells has demonstrated a reduction in intestinal tumor burden [208, 209]. Furthermore, the Wnt/β-catenin signaling pathway exhibits crosstalk with other pathways and actively participates in the regulation of tumor microenvironment [210, 211]. Dysregulated Wnt signaling orchestrates intricate interactions between transformed cells and infiltrating immune cells, thereby fostering immune tolerance and constraining anti-tumor responses [212]. Additionally, cancer-associated fibroblasts (CAF) can enhance invasion and migration of colorectal cancer cells by releasing Wnt2 protein [213]. The RSPO family comprises a group of secreted proteins synthesized by stromal cells within the tumor microenvironment that augment Wnt signaling [214].

##### Hepatocellular carcinoma

HCC is one of the most prevalent carcinomas worldwide and a leading cause of cancer-related death [215]. The CTNNB1 mutation represents one of the most crucial genetic events in human HCC, characterized by an increased prevalence of β-catenin overexpression and mutations in hepatitis C virus related HCC compared to hepatitis B virus related cases [216, 217]. Although β-catenin has been associated with early-stage HCC, its prognostic significance remains controversial [218]. Importantly, CTNNB1 mutation may impact the pathological presentation of HCC. Audard et al. demonstrated that CTNNB1-mutated HCCs typically manifest as large solitary lesions (> 6 cm in diameter) [219]. The activation of Wnt/β-catenin alone is insufficient to drive hepatocarcinogenesis; instead, its interactions with c-Met, K-RasV12, activated Akt, LKB1, and Nrf2 are necessary for HCC formation in mice. c-Met has been observed in 10% of human HCC tissue samples [220–224]. AXIN1 also plays a crucial role in HCC genesis. In AXIN1-deleted mice, 40% of mutated mice developed HCC, while none of the control monogenic mice exhibited HCC development [225]. The deletion of AXIN1 also triggers the activation of Notch and YAP pathways, synergistically contributing to HCC progression [225]. Numerous regulators associated with the Wnt signaling pathway have been investigated in HCC. For example, peroxiredoxin 2 (PRDX2) exhibits the capability of Wnt/β-catenin pathway activation by promoting β-catenin nuclear translocation. It has been identified that in patients with HCC, PRDX2 levels in bile are significantly higher compared to those with choledocholithiasis. Evidence suggests that silencing PRDX2 results in the induction of senescence in HCC cells [226].

##### Breast cancer

Abnormalities in the Wnt signaling pathway that induce breast cancer development are implicated at various levels, encompassing DNA, mRNA, protein, and subcellular localization. Analogous to other tumors, activation of Wnt signaling frequently transpires in breast cancer. Deletion, mutation, reduction or hypermethylation of DC components commonly occur, thereby facilitating the nuclear entry of β-catenin proteins. The aberrant activation of the Wnt/catenin pathway in breast cancer is predominantly induced by epigenetic alterations in constituent elements, such as C-terminal binding protein [227] and Groucho-related transcriptional repressor protein [228]. Invasive ductal carcinoma (IDC) and invasive lobular carcinoma (ILC) are two prevalent subtypes of breast cancer associated with poor prognosis. IDC demonstrates consistent expression of β-catenin, both in the membrane and nucleus [229]. In contrast, ILC does not exhibit these expressions and, in cases of lobular carcinoma in situ, also lacks expression of the E-cadherin protein [230]. In brief, abnormalities in the Wnt signaling pathway play a critical role in breast carcinogenesis, and therefore necessitate thorough and meticulous investigation [231]. In breast cancer, the expression of β-catenin mRNA is significantly upregulated in ALDH ^hi^ CD44^+^ breast CSCs and positively correlates with Ki67 expression. This suggests a close association between β-catenin and the self-renewal function of breast CSCs [232]. Meanwhile, aggregated β-catenin can promote the expression of plasma protease (MMP-9, MMP-12), thereby enhancing the resistance and invasiveness of tumor cells [232]. Notably, CSCs exhibit higher Wnt/β-catenin signaling compared to other cancer cell types in breast cancer. Treatment with CWP232228, a β-catenin inhibitor that antagonizes the binding of β-catenin to TCF in the nucleus, effectively reduces intracellular Wnt3a transcriptional activity and inhibits CSC proliferation. Therefore, this study suggests that targeting β-catenin may hold significant therapeutic potential for addressing cancer metastasis, recurrence, and drug resistance in breast cancer associated with CSCs [233].

##### Prostate cancer

β-catenin proteins play an indispensable role in embryonic prostate development, whereas its overexpression contributes to invasive prostate cancer development [234]. The increased levels of β-catenin proteins in prostate tumor cells primarily result from gene mutations and alterations in the expression of activators and inhibitors within the Wnt signaling pathway [235]. Although genetic and epigenetic changes activate Wnt/β-catenin signaling to drive the progression of prostate cancer [235], it is not commonly observed for CTNNB1, APC, and AXIN1 genes to undergo mutations in prostate cancer [236]. It is noteworthy that activation of Wnt/β-catenin signaling due to genetic changes occurs more frequently in castration-resistant prostate cancer (CRPC) compared to treatment-naïve cases, as evidenced by clinical samples obtained from CRPC patients, which demonstrate alterations in CTNNB1 and APC genes [237]. Moreover, APC deletion in combination with the prostate oncogene Hepsin overexpression facilitates prostate cancer progression [238]. Mouse models of prostate cancer can be generated by inducing stable forms of β-catenin or APC deletion [235]. The secretion of Wnt by prostate stromal cells significantly influences tumorigenesis and progression [235]. For instance, Wnt3a has been certified to activate canonical Wnt signaling in epithelial cells, thereby promoting the progression of high-grade prostate intraepithelial neoplasia to adenocarcinoma [174]. Additionally, Wnt16b, another member of the Wnt family, activates the classical Wnt program within tumors in a paracrine manner. Consequently, this mechanism diminishes the efficacy of chemotherapy in eradicating cancer cells and fosters the development of drug resistance [239]. Targeting the Wnt receptor on the cell membrane or inhibiting the function of β-catenin-associated proteins represents an effective approach for prostate cancer treatment.

##### Melanoma

The Wnt signaling pathway governs the migration of neural crest melanocytes to the hair and epidermal follicles, where they facilitate cellular differentiation and generate skin and hair pigment [240]. Activation of this pathway leads to an increase in the melanocyte population as well as a decline in the number of neurons and glial cells [240]. In terms of melanocyte development, the Wnt signaling pathway assumes a pivotal role by activating the microphthalmia-associated transcription factor, which regulates cell survival, proliferation, and differentiation processes [241]. Additionally, the activation of the Wnt signaling pathway in melanoma is known to occur through the ubiquitination and degradation of CD44 and cortactin by RNF128, subsequently inducing EMT and stemness in melanoma cells. This process is considered to promote melanoma progression and is significantly associated with a poor prognosis in melanoma patients [242]. The role of β-catenin in the later stages of melanoma development remains controversial, as some studies propose that elevated levels of β-catenin in melanoma are associated with a lower proliferative index [243] or a better prognosis [244]. However, numerous studies have demonstrated that the oncogenic role of β-catenin in melanoma [245, 246]. Hence, further investigation is imperative to fully comprehend the dynamic involvement of β-catenin proteins in melanoma progression, which can provide valuable insights into its pathogenesis and guide the development of targeted therapies. It is widely postulated that distinct Wnt signaling pathways are harnessed by melanoma cells at different stages of disease advancement; canonical Wnt signaling predominantly facilitates growth and transformation to augment their proliferative capacity [247, 248].

##### Renal cell carcinoma

In numerous patients with renal cell carcinoma (RCC), various abnormalities have been observed in the expression of Wnt proteins, Wnt receptors, and Wnt antagonists. Some of these aberrations include upregulation of Wnt1 [249] and Wnt10a [250], as well as downregulation of Wnt5a [251] and Wnt7a [252]. In addition, RCC also exhibits elevated mRNA levels of Wnt receptors FZD5 and FZD8, resulting in increased cyclin D1 [253]. The elevated protein expression level of FZD7 and restoration of FZD7 function effectively reverses the inhibitory effect exerted by the tumor suppressor miR-613 on RCC cell proliferation and invasion [254]. Alterations in β-catenin expression have also been observed in RCC [255]. Cytoplasmic accumulation of β-catenin is considered a promising therapeutic approach due to their potential role in maintaining cellular homeostasis [256]. The Wnt signaling pathway is tightly regulated by Wnt antagonists. Any attenuation or loss of these regulators can result in further aberrations within the pathway. These aberrations encompass the down-regulation of sFRP members, the DKK family, and WIF1, among others [257]. Guo et al. discovered a significant decrease in serum levels of DKK1 and DKK3 among ccRCC patients compared to healthy individuals [258]. The reduced levels of DKK1 might be attributed to various factors including promoter hypermethylation and histone modifications. DKK1 is an inhibitor of the Wnt signaling pathway while DKK3 is generally considered to act as a tumor suppressor [259]. It has been suggested that there is an interactive relationship between DKK1 and DKK3 [258]. Furthermore, although the canonical Wnt signaling pathway has been extensively studied, further investigation is required to understand the non-canonical Wnt pathway in RCC. Gaining insights into the intricate interactions between the Wnt pathway and other pathways could facilitate the identification of novel therapeutic targets and more effective diagnostic markers.

##### Glioblastoma

Active Wnt/β-catenin signaling has been correlated with diminished survival rates among patients diagnosed with GBM. Although abnormalities in major components of the Wnt pathway are infrequently observed in GBM [260], APC mutations have been found to occur in approximately 13% of GBM patients, with a frequency of around 14.5% [261]. Increased binding of cadherin to α- or β-catenin promotes the migration of glioma cells and EMT [261]. Additionally, phosphorylation of β-catenin stimulates the migration and activation of glioma cells. Wnt5a modulates the synthesis of matrix metalloproteinase-2 (MMP-2), thereby enhancing glioma cell migration [262]. Autophagy, a crucial cellular process involving the degradation of cytoplasmic components by lysosomes, plays a significant role in attenuating Wnt signaling in GBM and inducing the intracellular relocalization of β-catenin proteins. It has been demonstrated robust autophagy can result from nutrient deficiency or the administration of mTOR inhibitors in GBM cells [260]. GSK3β promotes autophagy by phosphorylating the tuberous sclerosis complex [260]. Mesenchymal stem cells (MSCs), a type of pluripotent stem cells derived from the mesoderm, possess robust migratory capacity and resistance to genotoxic substances attributed to the EMT undergone by polarized epithelial cells [261, 263]. GBM with mesenchymal features is characterized by a high degree of aggression and resistance towards treatment along with neural stem cell marker [264]. Adaptive radio-resistance in patients with GBM is regulated by a diverse range of proteins, including N-cadherin and β-catenin. The concurrent expression of N-cadherin and β-catenin leads to the attenuation of Wnt/β-catenin signaling, consequently inhibiting the proliferation of neuronal cells. Moreover, reduced levels of N-cadherin and β-catenin have been found to enhance cellular sensitivity to radiation therapy [261].

##### Osteosarcoma

Osteosarcoma (OS) is a disease characterized by aberrant cellular differentiation, which arises from the transformation of pluripotent MSCs [265]. In mice, the absence of the CDKN2A locus promotes the formation of OS [266]. Human OS cells possessing MSC characteristics exhibit distinct genetic profiles compared to other tumor cell types, enabling them to maintain an undifferentiated state [267]. Numerous studies have demonstrated that dysregulated activation of the canonical Wnt signaling pathway contributes to both the development and metastasis of OS [268, 269]. It has been observed that OS tissues exhibit elevated levels of β-catenin compared to adjacent healthy tissues, resulting in poor prognosis and the occurrence of lung metastasis [270]. The inactivation of the Wnt/β-catenin pathway, particularly through the deletion of genes associated with this signaling pathway, such as c-myc and cyclin D1 proteins, also contributes to the development of OS [271]. Similarly, OS is characterized by the presence of EMT, which inhibits key components of intercellular junctions, including E-cadherin, thereby promoting the invasiveness of cancer cells [272]. Furthermore, a study has demonstrated that Echinatin (Ecn), a naturally occurring active compound, exerts inhibitory effects on the proliferation, migration, and invasion of OS cells by suppressing the Wnt/β-catenin signaling pathway while simultaneously activating the p38 signaling pathway [273]. Long non-coding RNA urothelial cancer-associated 1 (UCA1) participates in OS pathogenesis by modulating the Wnt/β-catenin pathway through the miR-145/HMGA1 axis. Inhibition of UCA1 and upregulation of miR-145 both impede the adverse progression of OS, suggesting that UCA1 holds the potential to be a therapeutic target for OS [274]. The activity of the Wnt pathway is closely tied to bone development and growth, and its dysregulation can lead to corresponding pathological conditions [275]. In conventional high-grade OS, the Wnt/β-catenin pathway is inhibited while reactivation of this pathway has been demonstrated to suppress cancer cell proliferation [276].

## Therapeutic targeting of the Wnt/β-catenin signaling pathway

**Fig. 3:**
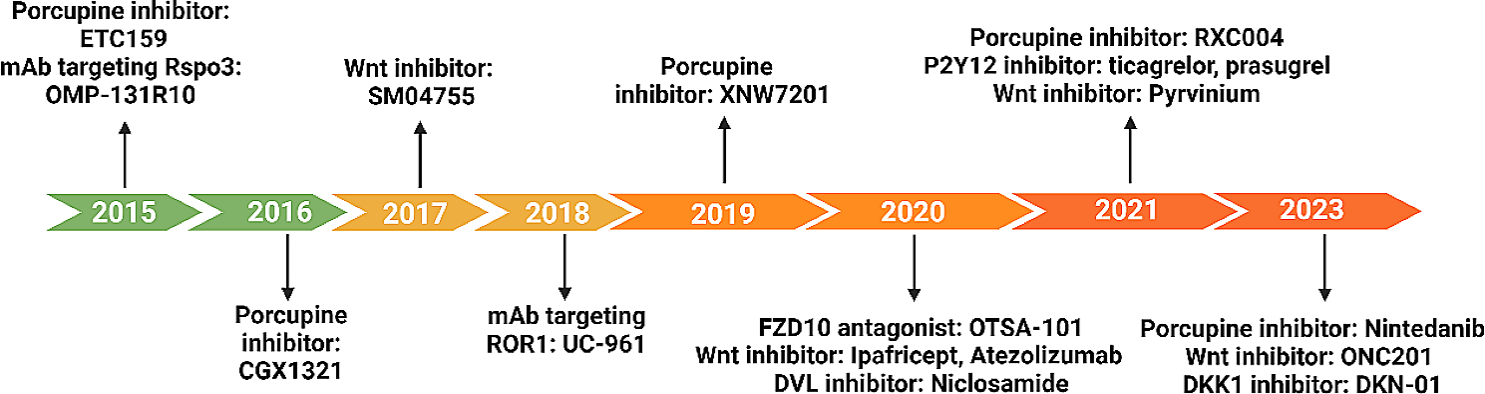
Timeline of clinical trials for agents targeting the Wnt/β-catenin signaling pathway

Abnormal activation of the Wnt/β-catenin signaling pathway is involved in various diseases and the oncogenic transformation of tumors, underscoring the significance of pivotal factors involved in Wnt/β-catenin signal transduction as promising targets for therapy. So far, numerous inhibitors targeting the Wnt/β-catenin signaling pathway have emerged as promising therapeutic agents for cancer treatment and are currently undergoing preclinical or clinical research. These drugs exhibit the capacity to effectively target specific components of the classical Wnt signaling cascade, leading to the attenuation of Wnt signal transduction and impeding cancer progression. The judicious selection of either Wnt activators or inhibitors is pivotal and should be based on critical factors such as the precise disease type, stage of advancement, and lesion characteristics. Extensive investigation into the targeted therapy against the Wnt/β-catenin pathway has yielded the identification and exploration of several inhibitors in clinical research. Clinical trials of agents targeting the Wnt–β-catenin signaling pathway are presented in Table 4; Fig. 3.

**Table 4:** Clinical trials of agents targeting Wnt–β-catenin signaling pathway

Name	Type	Target	Function	Cancer types	Phase	Clinical trial record
Wnt974	SMI	Porcupine	Inhibition of Wnt lipid modifications	Metastatic Colorectal Cancer	I/II	NCT02278133
RXC004/0001	SMI	Porcupine	Inhibition of Wnt lipid modifications	Advanced cancer	I/IIa	EUCTR2017-000720-9
XNW7201	SMI	Not report	A Wnt pathway inhibitor	Advanced Solid Tumors	I	NCT03901950
OMP-54F28	SMI	FZD8	Decoy receptor for Wnt ligands	Solid Tumors	I	NCT01608867
PRI-724	SMI	CREB-binding protein	Blocking the Wnt signaling pathway	Acute Myeloid Leukemia	I/II	NCT01606579
FOG-001	SMI	β-catenin	A Wnt pathway inhibitor	Colorectal Cancer	I/II	NCT05919264
Sinecatechins	Natural ingredient	Not report	Inactivation of β-catenin signaling	Carcinoma Basal Cell	II/III	NCT02029352
RNA circ-ITCH	Natural ingredient	Not report	Inhibiting Wnt/ß-catenin signaling pathway	Colon cancer	NR	ChiCTR2100048989
Micro RNA-506	Natural ingredient	Not report	Down-regulating LHX2	Nasopharyngeal carcinoma	NR	ChiCTR1800018889
Calcitriol	Natural ingredient	Wnt/β-catenin	A Wnt pathway inhibitor	Basal cell cancer	I	NCT01358045
Resveratrol	Natural ingredient	Not report	Resveratrol modulates Wnt signaling	Colon Cancer	I	NCT00256334
PTK7-ADC	Antibody	Not report	A Wnt pathway inhibitor	Triple Negative Breast Cancer	I	NCT03243331
DKN-01	Antibody	DKK1	Wnt signaling Alterations - DKN-01 inhibition	Hepatocellular Carcinoma	I/II	NCT03645980
Cirmtuzumab	Antibody	NR	A Wnt pathway inhibitor	Metastatic Castration-resistant Prostate Cancer	I	NCT05156905

**Fig. 4:**
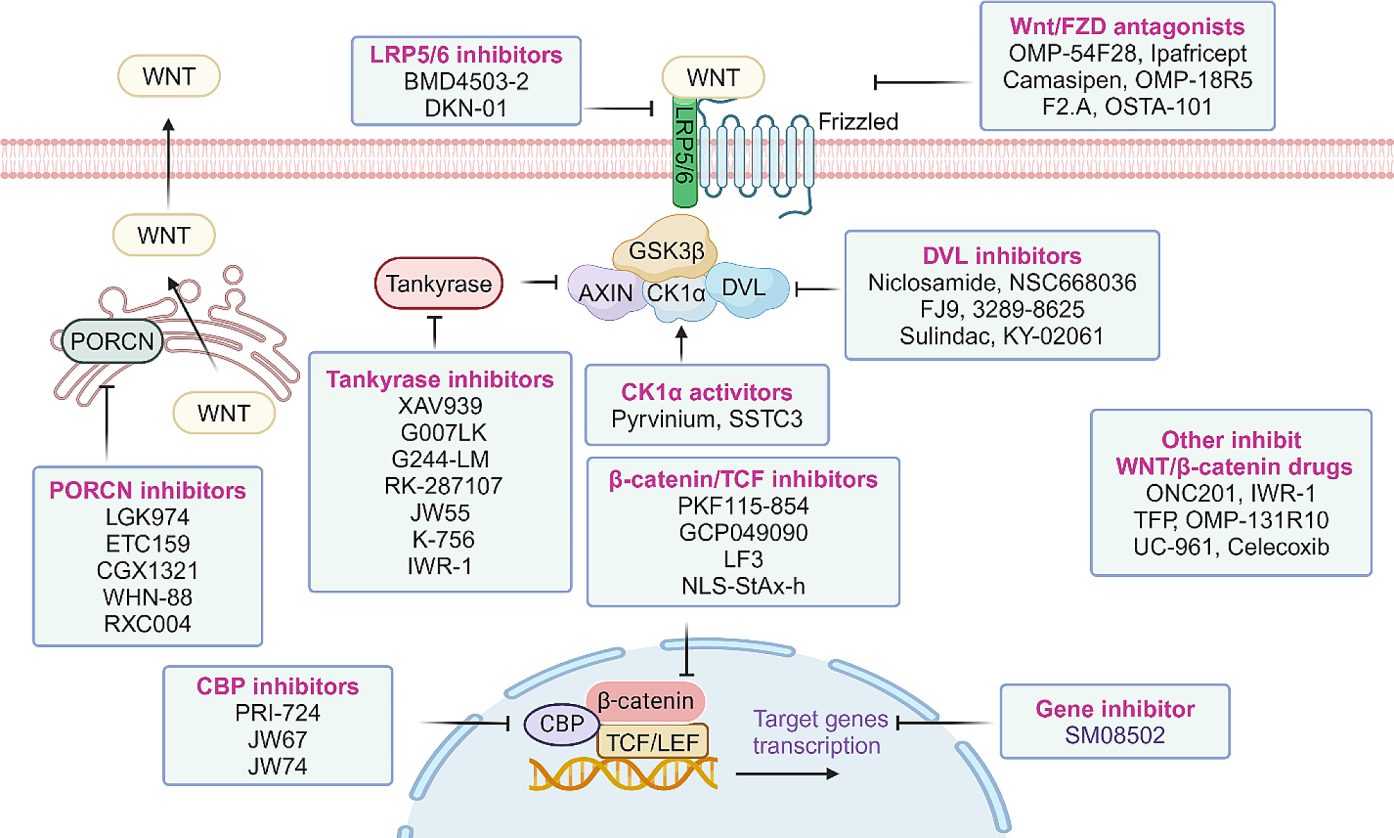
Therapeutic strategies targeting Wnt/β-catenin signaling pathway. Currently, many Wnt/β-catenin signaling pathway inhibitors have become potential anti-cancer therapeutic drugs and are currently undergoing preclinical or clinical research. These drugs work by targeting specific components of the canonical Wnt signaling pathway, causing downregulation of Wnt signaling and inhibiting cancer progression. The selection of Wnt activators or inhibitors is crucial. In terms of specific target components within the pathway, these drugs can be divided into the following categories: (1) Extracellular or membrane level inhibitors or antagonists, which target Wnt. Ligands: FZD, LRP5/6 (2). Cytoplasmic level inhibitors, targeting β-catenin, AXIN, APC and other components involved in pathway activation; (3) Nuclear level inhibitors, regulating the transcription of target genes, such as transcriptional co-factors; (4) Inhibitors that interact with other signaling pathways

The targeted agents of the Wnt/β-catenin pathway can be classified into various types based on different dimensions. In terms of the specific components targeted within the pathway, these agents can be categorized as follows: (1) extracellular or membrane level inhibitors, which target the Wnt ligands, FZD, LRP5/6, WIF, DKK-1, and other secreted soluble regulators; (2) cytoplasmic level inhibitors, which target β-catenin, AXIN, APC, and other components involved in pathway activation; (3) nuclear level inhibitors, which modulate the transcription of transcription of target genes, such as transcriptional co-factors; and (4) inhibitors that interact with other signaling pathways [315]. Additionally, these agents can be further classified based on their nature and application, including pharmaceuticals/phytochemicals, Wnt inhibitory molecules, or clinical Wnt inhibitors that specifically target the Wnt pathway. Given the significant association of this pathway with carcinogenesis, extensive research has been conducted in this field. The mechanism of these targeted agents is illustrated in Fig. 4.

### Small-molecule inhibitor

Small-molecule inhibitors (SMIs) are commonly recognized as chemically synthesized compounds with a molecular weight below 1 kDa [326]. Due to their lower cost, facile manufacturing process, oral bioavailability, and ability to target both extracellular and intracellular components, SMIs possess indispensable potential. These inhibitors can be classified into three categories: small molecules that target cytoplasmic proteins, those targeting transcriptional factors, and those targeting co-activators [277]. The SMIs targeting the Wnt/β-catenin pathway are summarized in Table 5.

#### Porcupine inhibitors

Inhibitors targeting PORCN have the ability to effectively suppress the post-translational acylation process of Wnt ligands, thereby impeding their secretion [278]. Among these inhibitors, Wnt974 (LGK974) particularly noteworthy for its demonstrated efficacy in various tumor models including mouse mammary tumor virus-Wnt-1 (MMTV-Wnt1) mouse model and models of human head and neck squamous cell carcinoma [279, 280]. It demonstrates remarkable efficacy in metastatic CRC with Wnt pathway mutations and head and neck squamous cell carcinoma with Notch mutations, as both tumor types exhibit high sensitivity to LGK974 [278, 280]. In vitro studies have also revealed the ability of LGK974 to diminish the viability of epithelial ovarian cancer (EOC) cells and impede tumor growth in vivo [281]. ETC159 is an orally available PORCN inhibitor that effectively obstructs the secretion and activity of all Wnts. It has exhibited significant therapeutic effects in xenografts derived from CRC patients harboring RSPO translocations [282]. CGX1321, another PORCN inhibitor, has been demonstrated to reduce tumor cell burden, enhance immune cell infiltration, and improve survival rate in an EOC mouse model [283]. WHN-88, a recently developed PORCN inhibitor, effectively impedes the palmitoylation of Wnt ligands, thereby obstructing their secretion as well as the Wnt/β-catenin signaling pathway activation [284]. This inhibition has demonstrated the ability to suppress mouse mammary tumor growth and impede Wnt-driven human tumor progression in xenografts. Other PORCN small molecule inhibitors, including IWP compounds, RXC004, and Wnt-C59, have been identified for their ability to block Wnt secretion by inhibiting the acylation of Wnt proteins [285–287]. Notably, the combination of ETC-159 (a PORCN inhibitor) and GDC-0941(a PI3K inhibitor) has demonstrated remarkable inhibition of proliferation in RNF43-mutant pancreatic cancer cells and effectively suppressed the growth of xenografts in vivo [288]. Furthermore, GNF-6231 has exhibited significant inhibitory effects on breast cancer in a mouse model [289].

#### FZD antagonists

The identification of selective inhibitors for FZD7 has posed a significant challenge. The Wnt-FZD pathway is activated by Wnt proteins, which are promiscuous ligands capable of binding to multiple FZDs [290]. In vitro studies have demonstrated that SRI37892, which interacts with the putative binding site on the transmembrane domain of FZD7, effectively inhibits cell proliferation and the β-catenin-dependent Wnt pathway in triple-negative breast cancer cells. Additionally, computational docking has identified ZINC05972969 as another small molecule that targets FZD7 [290]. Niclosamide facilitates the endocytosis of FZD1, leading to the downregulation of Wnt3a-induced β-catenin stabilization [291].

#### LRP5/6 inhibitors

BMD4503-2, a quinoxaline derivative, has been identified as a novel small-molecule inhibitor that blocks the interaction between LRP5/6 and sclerostin. Through competitive binding to the LRP5/6-sclerostin complex, BMD4503-2 has the potential to restore the decreased activity of the Wnt/β-catenin signaling pathway [292]. Mesd, a universal inhibitor of LRP5/6 ligands, plays a dual role in inhibiting the binding of the Wnt antagonists DKK1 and Sclerostin to LRP5/6 receptors, as well as suppressing Wnt/β-catenin signaling induced by Wnt3a and Rspondin1 in Lrp5/6-expressing cells. Furthermore, Mesd demonstrates the ability to inhibit LRP6 phosphorylation and Wnt/β-catenin signaling in prostate cancer PC-3 cells, resulting in the suppression of PC-3 cell proliferation [293]. Niclosamide, calcipotriol, Salinomycin, pantoprazole and monensin downregulates the total and p-LRP6, leading to its degradation and inhibition of Wnt/β-catenin signaling pathway [294, 295].

#### DVL inhibitors

The DVL protein family comprises three members: DVL-1, DVL-2, and DVL-3, which can bind to FZD and regulate small GTPases [34]. As an essential component in the Wnt/β-catenin signaling pathway, the DVL family represents an ideal target for anti-tumor treatment. DVL recruits the components of DC to the cell membrane by binding to the cytoplasmic carboxyl end of the FZD protein through its PDZ domain, thereby modulating the Wnt signaling pathway [41, 296]. Niclosamide, an antihelminthic compound, has demonstrated efficacy in promoting FZD endocytosis while downregulating DVL-2 and inhibiting β-catenin [297, 298]. NSC668036, FJ9, and 3289–8625 effectively block the interaction between DVL PDZ domain and FZD, subsequently inhibiting signal transduction pathways [299–302]. In addition, sulindac, as an SMI, effectively impedes the β-catenin signal transduction pathway triggered by Wnt3a at the DVL level [303]. Another noteworthy SMI, KY-02061, exhibits its potential to obstruct the interaction between DVL and CXXC5, which is involved in the negative feedback regulation of Wnt/β-catenin signal transduction [130].

#### SMIs targeting DC

Tankyrases (TNKS) function as inhibitory agents that specifically regulate the DC of β-catenin. AXIN, a rate-limiting scaffolding protein within the complex, is continuously regulated by TNKS [304–306]. TNKS primarily contribute to the degradation of AXIN protein, and its inhibition leads to the stabilization of AXIN, thereby antagonizing Wnt signaling [307]. Several TNKS inhibitors with favorable therapeutic effects have been developed, including XAV939, G007LK, G244-LM, RK-287,107, JW55, K-756, IWR-1, MSC2504877, AZ1366, JW74, and NVP-TNKS656 [308–317]. CK1α strictly modulates the canonical Wnt signal transduction and serves as a negative regulator of Wnt signaling, playing a crucial role in the formation of DC and subsequent phosphorylation of β-catenin [318]. Pyrvinium, an FDA-approved drug, has demonstrated efficacy against various cancers. It effectively stabilizes AXIN and facilitates β-catenin turnover, thereby inhibiting Wnt signal transduction [319]. Moreover, pyrvinium directly activates CK1α kinase activity by attenuating CRBN-mediated degradation of CK1α, further suppressing canonical Wnt signal transduction [320]. SSTC3, a small molecule activator of CK1α, can inhibit the growth of heterologous transplants in CRC [321, 322].

#### SMI targeting TCF

There are also small molecule inhibitors available that effectively disrupt the interaction between β-catenin and TCF. For example, the fungal derivative PKF115-854 and CGP04909036-40 exhibit dose-dependent inhibition of the β-catenin/TCF complex or hinder the binding of β-catenin to cAMP response element-binding protein (CREB)-binding protein [323–327]. PKF115-854 and CGP049090 have also presented inhibitory effects on HCC cell growth [327, 328]. Additionally, LF3, a derivative of the 4-thiouracil-benzene sulfonamide compound, significantly disrupts the crucial interaction between β-catenin and transcription factor TCF4. Furthermore, NLS-StAx-h, a selective cell-penetrating peptide inhibitor of β-catenin transcriptional factor interaction, effectively inhibits the proliferation and migration of colorectal cancer cells [329]. SM08502 is a small molecule inhibitor that targets the Wnt signaling pathway. By blocking the interaction between β-catenin and TCF/LEF, SM08502 disrupts the formation of the transcriptional complex required for Wnt target gene expression [330].

#### Transcription co-factor inhibitors

Multiple coactivators involved in Wnt/β-catenin transcription, including CBP/p300, BCL9, and pygopus, have been identified. In recent years, several SMIs targeting CBP, which acts as one of the coactivators for β-catenin-dependent transcription, have emerged and exhibited promising anti-tumor effects in preclinical models [331–333]. ICG-001 specifically disrupts the interaction between CBP and β-catenin, thereby inhibiting the transcriptional activation of Wnt target genes. Additionally, ICG-001 selectively target human CSCs, leading to the disruption of the CBP/β-catenin complex and the formation of a specific Sam68/CBP complex within the CSCs [334]. PRI-724 serves as a pioneer small molecule antagonist, similar to ICG-001 [335]. Upon phosphorylation, PRI-724 undergoes rapid hydrolysis in vivo to convert into its active form known as C-82 to inhibit the interaction between β-catenin and CBP [336]. In cases of chemotherapy-resistant EOC with overactivated CBP/β-catenin signaling pathway, PRI-724 has been found to enhance sensitivity of tumor to platinum chemotherapy while exhibiting significant toxicity in preclinical investigations [336, 337]. C646 is a potent and selective small molecule inhibitor of CBP’s histone acetyltransferase (HAT) activity. By inhibiting CBP’s HAT function, C646 blocks the acetylation of histones, leading to the repression of Wnt target gene transcription [338]. INT-001, a selective antagonist of CBP, demonstrated significant antitumoral activity in various preclinical models including pancreatic, colon, and tamoxifen-resistant breast cancers [339, 340].

#### Targeting CSCs

Several compounds targeting CSCs via the Wnt/β-catenin signaling pathway are commercially available. Wnt974 (LGK-974), a PORCN inhibitor, has demonstrated its ability to effectively impede the proliferation of breast CSCs. Chloroquine has been identified as an inhibitor of the Wnt/β-catenin pathway and demonstrates selective anti-tumor properties by targeting ovarian CSCs. Additionally, chloroquine reduces the levels of CSCs and their self-renewal ability in basal-like breast cancer through downregulation of LRP6 and β-catenin expression [341]. Furthermore, research has demonstrated that ONC201 significantly inhibits the expression of CSC-related genes in prostate tumors and GBMs by suppressing the Wnt/β-catenin pathway [342].

Some potential agents are currently undergoing preclinical evaluation for their ability to target CSCs by inhibiting the Wnt/β-catenin pathway. For instance, IWR-1, a tankyrase inhibitor, has been identified to impair the self-renewal of osteosarcoma CSCs and enhance their sensitivity to doxorubicin by affecting the intracellular translocation of β-catenin. Trifluoperazine, an antipsychotic drug, has also demonstrated inhibition of lung CSC sphere formation through suppression of Wnt/β-catenin signaling transduction and downregulation of lung CSC markers [341]. IC-2, a small molecule inhibitor, effectively diminishes the population of CD44 cells (liver CSCs) and impedes sphere-forming capacity in HCC, CRC, and bladder cancer cells [343–345]. Similarly, FH535 exhibits comparable effects by repressing the expression of liver CSC markers CD24 and CD44 [346]. These therapeutic approaches can be designed to specifically target the stemness of CSCs, thereby inhibiting tumor progression.

**Table 5:** Small-molecule compounds targeting Wnt/β-catenin pathway

Agents	Mechanism	Cancer type	Phase	Ref.
Wnt974 (LGK974)	Porcupine inhibitors	Breast cancer	Phase I	[279, 280]
ETC159	Porcupine inhibitors	Colorectal cancer, Osteosarcoma	Phase I	[282]
CGX1321	Porcupine inhibitors	Ovarian cancer	Phase I	[283]
WHN-88	Porcupine inhibitors	Pancreatic carcinoma	Preclinical	[284]
RXC004	Porcupine inhibitors	Colorectal cancer, Pancreatic cancer	Phase II	[286]
IWP compounds	Porcupine inhibitors	cancer	Preclinical	[283]
Wnt-C59	Porcupine inhibitors	Breast cancer	Preclinical	[283]
SRI37892	FZD7	triple-negative breast cancer	Preclinical	[291]
ZINC05972969	FZD7	Not determined	Preclinical	[290]
Niclosamide	FZD1	Glioblastoma cells	Preclinical	[298]
FJ9	DVL inhibitor	Lung Cancer, Lymphoma, Colorectal cancer, Melanoma	Preclinical	[302]
NSC668036	DVL inhibitor	Cervical cancer	Preclinical	[301]
3289–8625	DVL inhibitor	Prostate cancer	Preclinical	[302]
sulindac	DVL inhibitor	Colorectal cancer	Preclinical	[303]
XAV939	TNKS inhibitors	Renal cancer	Preclinical	[308]
G007LK	TNKS inhibitors	colorectal cancer	Preclinical	[309]
G244-LM	TNKS inhibitors	colorectal cancer	Preclinical	[310]
RK-287,107	TNKS inhibitors	Colorectal cancer	Preclinical	[311]
JW55	TNKS inhibitors	Colorectal cancer	Preclinical	[312]
K-756	TNKS inhibitors	Colorectal cancer	Preclinical	[313]
IWR-1	TNKS inhibitors	Lung cancer	Preclinical	[314]
MSC2504877	TNKS inhibitors	Colorectal cancer	Preclinical	[315]
AZ1366	TNKS inhibitors	Colorectal cancer	Preclinical	[314]
NVP-TNKS656	TNKS inhibitors	Colorectal cancer	Preclinical	[317]
Pyrvinium	CK1α activator	Colon cancer	Preclinical	[319]
SSTC3	CK1α activator	Colorectal cancer	Preclinical	[321, 322]
PKF115-854	β-catenin	Hepatocellular Carcinoma	Preclinical	[327, 328]
CGP04909036-40	β-catenin	Hepatocellular Carcinoma	Preclinical	[327, 328]
LF3	β-catenin	Colorectal cancer	Preclinical	[329]
NLS-StAx-h	β-catenin	Colorectal cancer	Preclinical	[329]
PRI-724	CREB-binding protein	Head and Neck Squamous Cancer Cells	Preclinical	[336, 337]
SM08502	Serine-arginine-rich splicing factor inhibitor	Hepatocellular Carcinoma	Preclinical	[330]
chloroquine	Cancer stem cells	Breast cancer, Ovarian cancer	Preclinical	[341]
ONC201	Cancer stem cells	Prostate cancer, glioblastomas	Preclinical	[342]
Trifluoperazine	Cancer stem cells	Lung cancer	Preclinical	[341]
IC-2	Cancer stem cells	Hepatocellular Carcinoma, Colorectal cancer, Bladder Cancer	Preclinical	[343–345]
FH535	Cancer stem cells	Colorectal cancer	Preclinical	[346]

### Monoclonal antibody

Monoclonal antibodies (mAbs) represent targeted therapeutics that exhibit significant therapeutic efficacy, particularly in the inhibition of extracellular or membrane-bound proteins. These antibodies are characterized by their high affinity and minimal off-target effects, as well as their extended plasma half-life and decreased clearance rate. mAbs not only directly interact with extracellular and membrane-bound targets but also activates immune responses to indirectly exert anti-tumor effects. In terms of direct action, mAbs bind to receptors or ligands to impede signal recognition or facilitate endocytosis, thereby diminishing the density of cell membrane surface receptors. Regarding indirect action, mAbs can induce complement-dependent cytotoxicity (CDC), antibody-dependent cellular cytotoxicity, or complement-dependent cytotoxicity mediated by cells [347].

#### mAbs targeting FZD

OMP-18R5, also known as vanticumab, is a mAb targeting human FZD1, 2, 5, 7, and 8 [231, 348]. Experimental evidence suggests its potential to inhibit the growth of gastric adenomas in mouse models irrespective of the presence of Apc LoF mutations. OMP-54F28, also known as ipafricept, represents a fusion protein integrating CRD of FZD8 and the immunoglobulin Fc domain [349]. By competitively binding to Wnt ligands, it negatively regulates the Wnt/β-catenin signaling transduction and disrupts the function of LRPs/FZDs [347, 350]. Ipafricept shares many similarities with vanticumab. Studies have shown that ipafricept exhibits significant anti-tumor effects in MMTV-Wnt1 cancer models. Additionally, the synergistic effect of ipafricept with gemcitabine has been demonstrated in pancreatic PDX models, significantly reducing the frequency of CSCs. This combination therapy strategy demonstrates superior tumor-blocking capabilities compared to the individual use of ipafricept or gemcitabine. Remarkably, both vanticumab and ipafricept exhibit remarkable synergistic effects when combined with taxanes (paclitaxel, cabazitaxel, and docetaxel). Fischer et al. have demonstrated the potential of pre-administering continuous ipafricept/vanticumab as a strategic approach to overcome resistance associated with taxanes alone, thus providing valuable insights for addressing this challenge in clinical practice. Mechanistically, the combination of ipafricept/vanticumab and taxanes augments cellular mitosis. The adverse reactions associated with OMPs resemble those of Porc inhibitors; however, OMPs exhibit more pronounced skeletal damage. Studies have indicated that vanticumab elicits more severe skeletal side effects in comparison with ipafricept. Overall, despite preclinical experiments demonstrating the favorable performance of OMPs, there remains a certain level of risk associated with their clinical applications.

F2.A is a newly developed antibody that selectively targets 6 out of 10 human FZDs (FZD1/2/4/5/7/8). The researchers initially identified an anti-FZD antibody (F2) with a specific profile that matched OMP-18R5. Subsequently, they employed combinatorial antibody engineering techniques to systematically explore and discover a variant (F2.A) with high specificity towards FZD4, thereby enabling the synthesis of this compound. Research findings have demonstrated that F2.A exhibits selective binding to FZD4 without competing with Norrin, another protein sharing a similar binding site. Moreover, in comparison with OMP-18R5 and OMP-54F28, F2.A demonstrates superior efficacy in treating RNF43-mutant pancreatic ductal adenocarcinoma.

OTSA-101, a humanized mAb targeting FZD10, has derivatives including 211At-OTSA-101, 111In-OTSA101, and 90Y-OTSA-101 [351]. Among them, 111In-OTSA-101 is commonly employed as a diagnostic tool. However, the antagonistic activity of OTSA-101 against the growth of synovial sarcomas cells is relatively weak. Experimental evidence has demonstrated that 211At-OTSA-101 tends to accumulate in the stomach and exhibits a lower uptake rate in tumor cells compared to 111In-OTSA-101. Nonetheless, 211At-OTSA-101 displays a more pronounced inhibitory effect than 90Y-OTSA-101. Furthermore, significant bone marrow suppression and notable hematological toxicity are observed with the administration of 90Y -OTSA − 101.

#### mAbs targeting RSPO3

OMP-131R10, also known as rosmantuzumab, is a humanized mAb targeting RSPO3. On the basis of the redundancy of RSPOs in activating Wnt/β-catenin signaling, certain malignant hematologic cancers exhibit aberrant activation of this pathway independent of Wnt ligands. Consequently, extensive research has demonstrated the ability of rosmantuzumab to impede self-renewal and differentiation processes in acute myeloid leukemia cells within PDX models, while simultaneously preserving the normal physiological function of hematopoietic stem cells [347].

#### mAbs targeting DKK1

DKN-01 is a humanized lgG4 mAb that can bind to and inhibit the activity of DKK1. The physiological mechanism associated with DKK1 involves the activation of DKK1 transcription through the Wnt/β-catenin signaling pathway, which then binds to LRP5/6 and blocks the recognition of Wnt ligands, thereby forming a negative feedback loop that inhibits Wnt signaling [352]. However, some tumors characterized by excessive Wnt/β-catenin signaling are not sensitive to DKK1. Excessive levels of DKK1 have been shown to facilitate tumor cell proliferation, but it is noteworthy that anti-DKK1 therapy may not only enhance Wnt signaling in osteoblasts but also in tumor cells. Although the precise underlying mechanism remains unclear, it is speculated that DKK1 contributes to tumor immune evasion. Experimental evidence has demonstrated that an intact immune system is necessary for the function of DKN-01, thereby exhibiting satisfactory tolerability in clinical trials and better efficacy in patients with DKK1 overexpression in particular. In murine studies, the administration of DKK1 mAb in combination with sclerostin antibody (Scl-mAb) at a low dose (3:1 dose of Scl-mAb: DKK1-mAb at12.5 mg/kg) has yielded significant skeletal benefits. These findings suggest that DKK plays a crucial role in negatively regulating bone mass in aging individuals [353].

### Natural bioactive compounds

In addition to synthetic compounds, an increasing number of natural products have been discovered to have an inhibitory effect on the Wnt/β-catenin protein signaling pathway. In comparison with conventional chemotherapy drugs, natural bioactive compounds typically exhibit minimal toxicity and can be readily obtained through dietary sources. These distinctive attributes render them promising adjunctive therapeutics for impeding cancer progression and mitigating the adverse effects associated with anticancer drugs.

Amongst the extensively investigated agents, resveratrol emerges as a prominent plant-derived compound that serves as a defensive mechanism against toxins in response to plant injury or pathogenic assault. Resveratrol has been found to inhibit the activation of β-catenin by preventing its nuclear translocation and subsequent interaction with TCF/LEF transcription factors. Resveratrol has been incorporated into two phase I clinical trials to investigate its potential in preventing CRC through dietary supplementation and reducing myocardial fibrosis [354]. Additionally, silybin, an extract derived from milk thistle seeds, significantly reduces the transcriptional activity of β-catenin-dependent TCF4 and the protein expression of β-catenin target genes. It has also demonstrated promising results in inhibiting tumor formation in preclinical models. Curcumin exhibits interference with the Wnt pathway and E-cadherin regulation, effectively suppressing the growth and invasion of CRC cells [355, 356]. Baicalein shows promise as well by inhibiting EMT, possibly through downregulation of SATB1 and the Wnt/β-catenin pathway. Moreover, flavonoids, a class of natural polyphenolic compounds, can exert preventive and anticancer effects on CRC by modulating the Wnt signaling pathway [210].

### Other potential compounds

Many drugs that have been approved by the FDA have expanded their indications leveraging their ability to interfere with the Wnt/β-catenin signaling pathway. Pyrvinium, an antiparasitic agent, effectively inhibits this pathway by activating CK1α. Niclosamide hinder canonical Wnt signaling through targeting components like DVL2 and LRP6 for degradation,while salinomycin specifically targets LRP6. Celecoxib promotes β-catenin degradation and induces TCF7 degradation by inhibiting prostaglandin E2 synthesisand cyclooxygenase activity of cyclooxygenase 2. Notably, chloroquine is an FDA-approved drug commonly utilized for treating tapeworm infection and stands out due to its mechanism involving internalization of the FZD1 receptor, reduction in DVL2 protein levels, and targeting of LRP6 to impede tumor proliferation, stemness, and metastasis [356, 357]. Piperazine, an anthelmintic agent, has been shown to inhibit the Wnt/β-catenin pathway through activation of CK1α or Gsk3β. Non-steroidal anti-inflammatory drugs have also been found to block cancer signaling pathways by inhibiting the activation of Wnt/β-catenin and reducing expression of its target genes via inhibition of β-catenin-mediated transcription [355]. Additionally, Cyclopamine has demonstrated anticancer activity through mediation of Wnt/β-catenin protein signaling transduction, resulting in inhibition of cell proliferation, induction of apoptosis, suppression of cell migration and invasion as well as angiogenesis and lymphangiogenesis.

## Prospects

With an increasing number of studies conducted on the Wnt/β-catenin signaling pathway, its pivotal role in carcinogenesis has gained universal recognition. Due to its intricately and delicately regulated nature, a multitude of regulators targeting the Wnt/β-catenin signaling pathway have been discovered, significantly advancing the development of therapeutic interventions. There are various approaches to Wnt/β-catenin targeted therapy. With the ongoing development of targeted drugs, preclinical research and clinical trials for intervening in Wnt/β-catenin signaling in malignant tumors have shown promising results, making them potential candidate drugs for personalized cancer treatment.

Nevertheless, despite numerous inhibitors for the activity of β-catenin have been identified, the majority have not achieved success in clinical practice. This suggests that targeting β-catenin still poses significant challenges in the context of cancer treatment. Firstly, due to the important role of the signaling pathway in normal cells, side effects may occur during the inhibitor treatment. As mentioned earlier, the Wnt/β-catenin signaling pathway plays a critical role in stem cell renewal and homeostasis, and targeted therapy may disrupt its normal function. Therefore, the clinical application of these therapies is limited [358, 359]. Moreover, long-term use of these drugs may lead to the progression of chronic diseases, such as chronic obstructive pulmonary disease. Meanwhile, tumor genesis is a complicated process that involves mutations and crosstalk between various signaling pathways. The understanding of the Wnt/β-catenin signaling pathway is still incomplete, and there are still many unknown mechanisms and regulatory factors. Therefore, it is necessary to comprehensively understand the specific role of Wnt/β-catenin in the occurrence and progression of diseases, to use these targeted drugs accurately and safely, ensuring their optimal application in clinical practice, and maximizing their therapeutic efficacy to benefit more patients.

## Conclusion

The Wnt/β-catenin signaling pathway plays a significant role in carcinogenesis through Wnt tumor suppressor mutations, epigenetic modifications such as DNA methylation and lysine acetylation, regulations of noncoding RNAs, and crosstalks with other signaling pathways. Therapies targeting the Wnt/β-catenin signaling pathway, such as SMIs, mAbs and natural bioactive compounds, have been extensively researched and utilized in clinical and preclinical studies. Nonetheless, it is important to note that blocking this pathway can disrupt tissue homeostasis and renewal, potentially leading to severe adverse events. Therefore, achieving a delicate balance between anti-tumor efficacy and minimizing adverse effects has become a crucial topic in current clinical trials. Additionally, the precise establishment of the signaling model is essential for further advancements in understanding this complex pathway. Despite these concerns, given the multitude of regulators involved and ongoing discoveries of novel components within the Wnt/β-catenin signaling pathway, it is highly likely that more effective therapies will be developed to enhance the prognosis of tumor patients.

## Data Availability

No datasets were generated or analysed during the current study.

## Abbreviations

APC: Adenomatous polyposis coli
BCL9: B-cell lymphoma 9
CAF: Cancer-associated fibroblasts
CBFβ: Core binding factor beta
CBP: CREB -binding protein
CDC: Complement-dependent cytotoxicity
CK1α: Casein kinase 1α
CLL: Chronic lymphocytic leukemia
CREB: cAMP response element-binding protein
CRPC: Castration-resistant prostate cancer
CRC: Colorectal carcinoma
CRD: Cysteine-rich domain
CSCs: Cancer stem cells
DC: Destruction complex
DKK: Dickkopf
DVL: Disheveled
Ecn: Echinatin
EMT: Epithelial-mesenchymal transition
EOC: Epithelial ovarian cancer
FAP: Familial adenomatous polyposis
FHA: Forkhead-associated domain
FOX: Forkhead box
FOXO: Forkhead box protein O
FZD: Frizzled
GBM: Glioblastoma
GPC: Glypican
GSK3β: Glycogen synthase kinase 3β
HCC: Hepatocellular carcinoma
HH: Hedgehog
HIF1α: Hypoxia-inducible factor 1α
IDC: Invasive ductal carcinoma
ILC: Invasive lobular carcinoma
KAT: Lysine acetyltransferase
KDAC: Catalytic function of lysine deacetylase
TCF/LEF: T cell-specific factor/Lymphoid enhancer-binding factor
LncRNAs: Long-chain non-coding RNAs
LRP5/6: Lipoprotein receptor 5/6
mAb: Monoclonal antibody
mCRPC: Metastatic castration resistant prostate carcinoma
MDR: Multi-drug resistance
miRNA: MicroRNA
MMP: Matrix metalloproteinase
MSC: Mesenchymal stem cell
MSI: Microsatellite instability
NSCLC: Non-small cell lung cancer
OS: Osteosarcoma
PORCN: Porcupine
PP1: Protein phosphatase 1
PTEN: Phosphatase and tensin homologue
PRDX2: Peroxiredoxin 2
RNF43: Ring finger protein 43
RSPO: Roof plate-specific spondin
RCC: Renal cell carcinoma
sFRPs: secreted frizzled-related proteins
SCFβ-TrCP: E3 ubiquitin ligase β-TrCP
SIRT: Sirtuin
SMIs: Small-molecule inhibitors
Smurf: Smad ubiquitin regulatory factor
SOX: Sex-determining region Y-box
TAZ: Transcriptional coactivator with PDZ-binding motif
TGF-β: Transforming growth factor-β
TNKS: Tankyrase
TSA: Traditional serrated adenoma
UCA1: Urothelial cancer-associated 1
WLS: Wntless
WIF: Wnt inhibitory factor
YAP: Yes-associated proteinZNRF3 Zinc and ring finger 3
ZNRF3: Zinc and ring finger 3

## References

[1] Salik B, Yi H, Hassan N, Santiappillai N, Vick B, Connerty P (2020). Targeting RSPO3-LGR4 signaling for leukemia stem cell eradication in Acute myeloid leukemia. Cancer Cell.

[2] Shen J, Sun Y, Liu X, Zhu Y, Bao B, Gao T (2021). EGFL6 regulates angiogenesis and osteogenesis in distraction osteogenesis via Wnt/β-catenin signaling. Stem Cell Res Ther.

[3] Choi BR, Cave C, Na CH, Sockanathan S (2020). GDE2-Dependent activation of canonical wnt signaling in neurons regulates oligodendrocyte maturation. Cell Rep.

[4] Wang Y, Krivtsov AV, Sinha AU, North TE, Goessling W, Feng Z (2010). The Wnt/beta-catenin pathway is required for the development of leukemia stem cells in AML. Science.

[5] Hawkins AG, Pedersen EA, Treichel S, Temprine K, Sperring C, Read JA et al. Wnt/β-catenin-activated ewing sarcoma cells promote the angiogenic switch. JCI Insight. 2020;5(13). 10.1172/jci.insight.135188 PMC7406270 32544094

[6] Unno K, Chalmers ZR, Pamarthy S, Vatapalli R, Rodriguez Y, Lysy B (2021). Activated ALK cooperates with N-Myc via Wnt/β-Catenin signaling to induce neuroendocrine prostate Cancer. Cancer Res.

[7] Mahmoudvand S, Shokri S, Taherkhani R, Farshadpour F (2019). Hepatitis C virus core protein modulates several signaling pathways involved in hepatocellular carcinoma. World J Gastroenterol.

[8] Wang J, Cai H, Liu Q, Xia Y, Xing L, Zuo Q (2020). Cinobufacini inhibits Colon Cancer Invasion and Metastasis via suppressing Wnt/β-Catenin signaling pathway and EMT. Am J Chin Med.

[9] Ghasemi F, Shafiee M, Banikazemi Z, Pourhanifeh MH, Khanbabaei H, Shamshirian A (2019). Curcumin inhibits NF-kB and Wnt/β-catenin pathways in cervical cancer cells. Pathol Res Pract.

[10] Sherman MH, Yu RT, Engle DD, Ding N, Atkins AR, Tiriac H (2014). Vitamin D receptor-mediated stromal reprogramming suppresses pancreatitis and enhances pancreatic cancer therapy. Cell.

[11] Dihlmann S, Siermann A, von Knebel Doeberitz M (2001). The nonsteroidal anti-inflammatory drugs aspirin and indomethacin attenuate beta-catenin/TCF-4 signaling. Oncogene.

[12] Li B, Cao Y, Meng G, Qian L, Xu T, Yan C (2019). Targeting glutaminase 1 attenuates stemness properties in hepatocellular carcinoma by increasing reactive oxygen species and suppressing Wnt/beta-catenin pathway. EBioMedicine.

[13] Cheng X, Xu X, Chen D, Zhao F, Wang W (2019). Therapeutic potential of targeting the Wnt/β-catenin signaling pathway in colorectal cancer. Biomed Pharmacother.

[14] Nusse R, Varmus HE (1982). Many tumors induced by the mouse mammary tumor virus contain a provirus integrated in the same region of the host genome. Cell.

[15] Kühl M, Sheldahl LC, Malbon CC, Moon RT (2000). Ca(2+)/calmodulin-dependent protein kinase II is stimulated by wnt and frizzled homologs and promotes ventral cell fates in Xenopus. J Biol Chem.

[16] Freisinger CM, Fisher RA, Slusarski DC (2010). Regulator of g protein signaling 3 modulates wnt5b calcium dynamics and somite patterning. PLoS Genet.

[17] Habas R, Dawid IB (2005). Dishevelled and wnt signaling: is the nucleus the final frontier?. J Biol.

[18] He X (2003). A wnt-wnt situation. Dev Cell.

[19] Zeng X, Tamai K, Doble B, Li S, Huang H, Habas R (2005). A dual-kinase mechanism for wnt co-receptor phosphorylation and activation. Nature.

[20] Willert K, Nusse R (2012). Wnt proteins. Cold Spring Harb Perspect Biol.

[21] Ke J, Xu HE, Williams BO (2013). Lipid modification in wnt structure and function. Curr Opin Lipidol.

[22] He X, Semenov M, Tamai K, Zeng X (2004). LDL receptor-related proteins 5 and 6 in Wnt/beta-catenin signaling: arrows point the way. Development.

[23] Alok A, Lei Z, Jagannathan NS, Kaur S, Harmston N, Rozen SG (2017). Wnt proteins synergize to activate β-catenin signaling. J Cell Sci.

[24] Komiya Y, Habas R (2008). Wnt signal transduction pathways. Organogenesis.

[25] Anastas JN, Moon RT (2013). WNT signalling pathways as therapeutic targets in cancer. Nat Rev Cancer.

[26] Ranes M, Zaleska M, Sakalas S, Knight R, Guettler S (2021). Reconstitution of the destruction complex defines roles of AXIN polymers and APC in β-catenin capture, phosphorylation, and ubiquitylation. Mol Cell.

[27] Nusse R, Clevers H (2017). Wnt/β-Catenin signaling, Disease, and emerging therapeutic modalities. Cell.

[28] Wiese KE, Nusse R, van Amerongen R. Wnt signalling: conquering complexity. Development. 2018;145(12). 10.1242/dev.165902 29945986

[29] Bilic J, Huang YL, Davidson G, Zimmermann T, Cruciat CM, Bienz M (2007). Wnt induces LRP6 signalosomes and promotes dishevelled-dependent LRP6 phosphorylation. Science.

[30] Kusserow A, Pang K, Sturm C, Hrouda M, Lentfer J, Schmidt HA (2005). Unexpected complexity of the wnt gene family in a sea anemone. Nature.

[31] Yu J, Chia J, Canning CA, Jones CM, Bard FA, Virshup DM (2014). WLS retrograde transport to the endoplasmic reticulum during wnt secretion. Dev Cell.

[32] Foord SM, Bonner TI, Neubig RR, Rosser EM, Pin JP, Davenport AP (2005). International Union of Pharmacology. XLVI. G protein-coupled receptor list. Pharmacol Rev.

[33] Gammons MV, Renko M, Johnson CM, Rutherford TJ, Bienz M (2016). Wnt Signalosome Assembly by DEP Domain Swapping of Dishevelled. Mol Cell.

[34] Dijksterhuis JP, Petersen J, Schulte G (2014). WNT/Frizzled signalling: receptor-ligand selectivity with focus on FZD-G protein signalling and its physiological relevance: IUPHAR Review 3. Br J Pharmacol.

[35] Li Y, Bu G (2005). LRP5/6 in wnt signaling and tumorigenesis. Future Oncol.

[36] Tamai K, Zeng X, Liu C, Zhang X, Harada Y, Chang Z (2004). A mechanism for wnt coreceptor activation. Mol Cell.

[37] Niehrs C, Shen J (2010). Regulation of Lrp6 phosphorylation. Cell Mol Life Sci.

[38] Brennan K, Gonzalez-Sancho JM, Castelo-Soccio LA, Howe LR, Brown AM (2004). Truncated mutants of the putative wnt receptor LRP6/Arrow can stabilize beta-catenin independently of frizzled proteins. Oncogene.

[39] Gao C, Chen YG, Dishevelled (2010). The hub of wnt signaling. Cell Signal.

[40] Zeng X, Huang H, Tamai K, Zhang X, Harada Y, Yokota C (2008). Initiation of wnt signaling: control of wnt coreceptor Lrp6 phosphorylation/activation via frizzled, dishevelled and axin functions. Development.

[41] Jiang X, Charlat O, Zamponi R, Yang Y, Cong F (2015). Dishevelled promotes wnt receptor degradation through recruitment of ZNRF3/RNF43 E3 ubiquitin ligases. Mol Cell.

[42] Nusse R, Clevers H (2017). Wnt/beta-Catenin signaling, Disease, and emerging therapeutic modalities. Cell.

[43] Wang Z, Li Z, Ji H (2021). Direct targeting of beta-catenin in the wnt signaling pathway: current progress and perspectives. Med Res Rev.

[44] Seidensticker MJ, Behrens J (2000). Biochemical interactions in the wnt pathway. Biochim Biophys Acta.

[45] Cadigan KM, Waterman ML. TCF/LEFs and wnt signaling in the nucleus. Cold Spring Harb Perspect Biol. 2012;4(11). 10.1101/cshperspect.a007906 PMC3536346 23024173

[46] Valenta T, Hausmann G, Basler K (2012). The many faces and functions of beta-catenin. EMBO J.

[47] Zhao H, Ming T, Tang S, Ren S, Yang H, Liu M et al. Wnt signaling in colorectal cancer: pathogenic role and therapeutic target. Mol Cancer. 2022;21(1). 10.1186/s12943-022-01616-7 PMC9281132 35836256

[48] Yan R, Fan X, Xiao Z, Liu H, Huang X, Liu J (2022). Inhibition of DCLK1 sensitizes resistant lung adenocarcinomas to EGFR-TKI through suppression of Wnt/beta-Catenin activity and cancer stemness. Cancer Lett.

[49] Wend P, Wend K, Krum SA, Miranda-Carboni GA (2012). The role of WNT10B in physiology and disease. Acta Physiol (Oxf).

[50] Chong JM, Uren A, Rubin JS, Speicher DW (2002). Disulfide bond assignments of secreted frizzled-related protein-1 provide insights about Frizzled homology and netrin modules. J Biol Chem.

[51] Malinauskas T, Aricescu AR, Lu W, Siebold C, Jones EY (2011). Modular mechanism of wnt signaling inhibition by wnt inhibitory factor 1. Nat Struct Mol Biol.

[52] Waghmare I, Page-McCaw A. Regulation of wnt distribution and function by Drosophila glypicans. J Cell Sci. 2022;135(3). 10.1242/jcs.259405 PMC8918805 35112708

[53] Li N, Wei L, Liu X, Bai H, Ye Y, Li D (2019). A Frizzled-Like Cysteine-Rich Domain in Glypican-3 mediates wnt binding and regulates Hepatocellular Carcinoma Tumor Growth in mice. Hepatology.

[54] Zhang X, Abreu JG, Yokota C, MacDonald BT, Singh S, Coburn KL (2012). Tiki1 is required for head formation via wnt cleavage-oxidation and inactivation. Cell.

[55] Zhang X, MacDonald BT, Gao H, Shamashkin M, Coyle AJ, Martinez RV (2016). Characterization of Tiki, a New Family of wnt-specific metalloproteases. J Biol Chem.

[56] Flanagan DJ, Pentinmikko N, Luopajärvi K, Willis NJ, Gilroy K, Raven AP (2021). NOTUM from apc-mutant cells biases clonal competition to initiate cancer. Nature.

[57] Kakugawa S, Langton PF, Zebisch M, Howell S, Chang TH, Liu Y (2015). Notum deacylates wnt proteins to suppress signalling activity. Nature.

[58] He Z, Zhang J, Ma J, Zhao L, Jin X, Li H (2023). R-spondin family biology and emerging linkages to cancer. Ann Med.

[59] Kazanskaya O, Glinka A, del Barco Barrantes I, Stannek P, Niehrs C, Wu W (2004). R-Spondin2 is a secreted activator of Wnt/beta-catenin signaling and is required for Xenopus myogenesis. Dev Cell.

[60] Glinka A, Dolde C, Kirsch N, Huang YL, Kazanskaya O, Ingelfinger D (2011). LGR4 and LGR5 are R-spondin receptors mediating Wnt/β-catenin and Wnt/PCP signalling. EMBO Rep.

[61] de Lau W, Peng WC, Gros P, Clevers H (2014). The R-spondin/Lgr5/Rnf43 module: regulator of wnt signal strength. Genes Dev.

[62] Foulquier S, Daskalopoulos EP, Lluri G, Hermans KCM, Deb A, Blankesteijn WM (2018). WNT signaling in Cardiac and Vascular Disease. Pharmacol Rev.

[63] Hao HX, Xie Y, Zhang Y, Charlat O, Oster E, Avello M (2012). ZNRF3 promotes wnt receptor turnover in an R-spondin-sensitive manner. Nature.

[64] Chang TH, Hsieh FL, Zebisch M, Harlos K, Elegheert J, Jones EY. Structure and functional properties of Norrin mimic wnt for signalling with Frizzled4, Lrp5/6, and proteoglycan. Elife. 2015;4. 10.7554/eLife.06554 PMC4497409 26158506

[65] Ke J, Harikumar KG, Erice C, Chen C, Gu X, Wang L (2013). Structure and function of Norrin in assembly and activation of a frizzled 4-Lrp5/6 complex. Genes Dev.

[66] Niehrs C (2006). Function and biological roles of the Dickkopf family of wnt modulators. Oncogene.

[67] Nakamura T, Nakamura T, Matsumoto K (2008). The functions and possible significance of Kremen as the gatekeeper of wnt signalling in development and pathology. J Cell Mol Med.

[68] Green J, Nusse R, van Amerongen R. The role of Ryk and Ror receptor tyrosine kinases in wnt signal transduction. Cold Spring Harb Perspect Biol. 2014;6(2). 10.1101/cshperspect.a009175 PMC3941236 24370848

[69] Mao B, Wu W, Davidson G, Marhold J, Li M, Mechler BM (2002). Kremen proteins are Dickkopf receptors that regulate Wnt/beta-catenin signalling. Nature.

[70] Niida A, Hiroko T, Kasai M, Furukawa Y, Nakamura Y, Suzuki Y (2004). DKK1, a negative regulator of wnt signaling, is a target of the beta-catenin/TCF pathway. Oncogene.

[71] Wang W, Li X, Lee M, Jun S, Aziz KE, Feng L (2015). FOXKs promote Wnt/β-catenin signaling by translocating DVL into the nucleus. Dev Cell.

[72] Prunier C, Hocevar BA, Howe PH (2004). Wnt signaling: physiology and pathology. Growth Factors.

[73] Jiang Y, Luo W, Howe PH (2009). Dab2 stabilizes Axin and attenuates Wnt/beta-catenin signaling by preventing protein phosphatase 1 (PP1)-Axin interactions. Oncogene.

[74] Li X, Yost HJ, Virshup DM, Seeling JM (2001). Protein phosphatase 2A and its B56 regulatory subunit inhibit wnt signaling in Xenopus. Embo j.

[75] Kamachi Y, Ogawa E, Asano M, Ishida S, Murakami Y, Satake M (1990). Purification of a mouse nuclear factor that binds to both the A and B cores of the polyomavirus enhancer. J Virol.

[76] Sweeney K, Cameron ER, Blyth K (2020). Complex interplay between the RUNX Transcription Factors and Wnt/β-Catenin pathway in Cancer: a Tango in the night. Mol Cells.

[77] Xiong J, Feng Z, Li Z, Zhong T, Yang Z, Tu Y (2019). Overexpression of TWA1 predicts poor prognosis in patients with gastric cancer. Pathol Res Pract.

[78] Lu Y, Xie S, Zhang W, Zhang C, Gao C, Sun Q (2017). Twa1/Gid8 is a β-catenin nuclear retention factor in wnt signaling and colorectal tumorigenesis. Cell Res.

[79] Ji L, Lu B, Wang Z, Yang Z, Reece-Hoyes J, Russ C (2018). Identification of ICAT as an APC inhibitor, revealing wnt-dependent inhibition of APC-Axin Interaction. Mol Cell.

[80] Lu L, Gao Y, Zhang Z, Cao Q, Zhang X, Zou J (2015). Kdm2a/b lysine demethylases regulate canonical wnt signaling by modulating the Stability of Nuclear β-Catenin. Dev Cell.

[81] Zhou Y, Xu J, Luo H, Meng X, Chen M, Zhu D (2022). Wnt signaling pathway in cancer immunotherapy. Cancer Lett.

[82] Goss KH, Groden J (2000). Biology of the adenomatous polyposis coli tumor suppressor. J Clin Oncol.

[83] Zhang L, Shay JW. Multiple roles of APC and its therapeutic implications in colorectal cancer. J Natl Cancer Inst. 2017;109(8). 10.1093/jnci/djw332 PMC5963831 28423402

[84] Bienz M, Hamada F (2004). Adenomatous polyposis coli proteins and cell adhesion. Curr Opin Cell Biol.

[85] Steigerwald K, Behbehani GK, Combs KA, Barton MC, Groden J (2005). The APC tumor suppressor promotes transcription-independent apoptosis in vitro. Mol cancer Research: MCR.

[86] Webster MT, Rozycka M, Sara E, Davis E, Smalley M, Young N (2000). Sequence variants of the axin gene in breast, colon, and other cancers: an analysis of mutations that interfere with GSK3 binding. Genes Chromosomes Cancer.

[87] Shimizu Y, Ikeda S, Fujimori M, Kodama S, Nakahara M, Okajima M (2002). Frequent alterations in the wnt signaling pathway in colorectal cancer with microsatellite instability. Genes Chromosomes Cancer.

[88] Jin LH, Shao QJ, Luo W, Ye ZY, Li Q, Lin SC (2003). Detection of point mutations of the Axin1 gene in colorectal cancers. Int J Cancer.

[89] Satoh S, Daigo Y, Furukawa Y, Kato T, Miwa N, Nishiwaki T (2000). AXIN1 mutations in hepatocellular carcinomas, and growth suppression in cancer cells by virus-mediated transfer of AXIN1. Nat Genet.

[90] Dahmen RP, Koch A, Denkhaus D, Tonn JC, Sörensen N, Berthold F (2001). Deletions of AXIN1, a component of the WNT/wingless pathway, in sporadic medulloblastomas. Cancer Res.

[91] Wu R, Zhai Y, Fearon ER, Cho KR (2001). Diverse mechanisms of beta-catenin deregulation in ovarian endometrioid adenocarcinomas. Cancer Res.

[92] Schaeffer S, Gupta B, Calatayud AL, Calderaro J, Caruso S, Hirsch TZ (2023). RSK2 inactivation cooperates with AXIN1 inactivation or β-catenin activation to promote hepatocarcinogenesis. J Hepatol.

[93] Belenguer G, Mastrogiovanni G, Pacini C, Hall Z, Dowbaj AM, Arnes-Benito R (2022). RNF43/ZNRF3 loss predisposes to hepatocellular-carcinoma by impairing liver regeneration and altering the liver lipid metabolic ground-state. Nat Commun.

[94] Hosein AN, Dangol G, Okumura T, Roszik J, Rajapakshe K, Siemann M (2022). Loss of Rnf43 accelerates Kras-mediated neoplasia and remodels the Tumor Immune Microenvironment in pancreatic adenocarcinoma. Gastroenterology.

[95] Eto T, Miyake K, Nosho K, Ohmuraya M, Imamura Y, Arima K (2018). Impact of loss-of-function mutations at the RNF43 locus on colorectal cancer development and progression. J Pathol.

[96] Neumeyer V, Brutau-Abia A, Allgäuer M, Pfarr N, Weichert W, Falkeis-Veits C (2021). Loss of RNF43 function contributes to gastric carcinogenesis by impairing DNA damage response. Cell Mol Gastroenterol Hepatol.

[97] Jiang X, Hao HX, Growney JD, Woolfenden S, Bottiglio C, Ng N (2013). Inactivating mutations of RNF43 confer wnt dependency in pancreatic ductal adenocarcinoma. Proc Natl Acad Sci U S A.

[98] Bao X, Zhang H, Wu W, Cheng S, Dai X, Zhu X et al. Analysis of the molecular nature associated with microsatellite status in colon cancer identifies clinical implications for immunotherapy. J Immunother Cancer. 2020;8(2). 10.1136/jitc-2020-001437 PMC7542666 33028695

[99] McMellen A, Woodruff ER, Corr BR, Bitler BG, Moroney MR. Wnt signaling in gynecologic malignancies. Int J Mol Sci. 2020;21(12). 10.3390/ijms21124272 PMC7348953 32560059

[100] Guo L, Wang X, Xu B, Lang R, Hu B (2021). Prognostic significance of CTNNB1 mutation in recurrence of sporadic desmoid tumors. Future Oncol.

[101] Björklund P, Lindberg D, Akerström G, Westin G (2008). Stabilizing mutation of CTNNB1/beta-catenin and protein accumulation analyzed in a large series of parathyroid tumors of Swedish patients. Mol Cancer.

[102] Liu Y, Patel L, Mills GB, Lu KH, Sood AK, Ding L et al. Clinical significance of CTNNB1 mutation and wnt pathway activation in endometrioid endometrial carcinoma. J Natl Cancer Inst. 2014;106(9). 10.1093/jnci/dju245 PMC4200060 25214561

[103] Ying J, Li H, Yu J, Ng KM, Poon FF, Wong SC (2008). WNT5A exhibits tumor-suppressive activity through antagonizing the Wnt/beta-catenin signaling, and is frequently methylated in colorectal cancer. Clin Cancer Res.

[104] Galamb O, Kalmar A, Peterfia B, Csabai I, Bodor A, Ribli D (2016). Aberrant DNA methylation of WNT pathway genes in the development and progression of CIMP-negative colorectal cancer. Epigenetics.

[105] Yoshikawa H, Matsubara K, Zhou X, Okamura S, Kubo T, Murase Y (2007). WNT10B functional dualism: beta-catenin/Tcf-dependent growth promotion or independent suppression with deregulated expression in cancer. Mol Biol Cell.

[106] Yu J, Tao Q, Cheng YY, Lee KY, Ng SS, Cheung KF (2009). Promoter methylation of the Wnt/beta-catenin signaling antagonist Dkk-3 is associated with poor survival in gastric cancer. Cancer.

[107] Yang B, Du Z, Gao YT, Lou C, Zhang SG, Bai T (2010). Methylation of Dickkopf-3 as a prognostic factor in cirrhosis-related hepatocellular carcinoma. World J Gastroenterol.

[108] He YH, Su RJ, Zheng J (2021). Detection of DKK-1 gene methylation in exfoliated cells of cervical squamous cell carcinoma and its relationship with high risk HPV infection. Arch Gynecol Obstet.

[109] Cui Y, Ma W, Lei F, Li Q, Su Y, Lin X (2016). Prostate tumour overexpressed-1 promotes tumourigenicity in human breast cancer via activation of Wnt/beta-catenin signalling. J Pathol.

[110] Suzuki H, Gabrielson E, Chen W, Anbazhagan R, van Engeland M, Weijenberg MP (2002). A genomic screen for genes upregulated by demethylation and histone deacetylase inhibition in human colorectal cancer. Nat Genet.

[111] Fujikane T, Nishikawa N, Toyota M, Suzuki H, Nojima M, Maruyama R (2010). Genomic screening for genes upregulated by demethylation revealed novel targets of epigenetic silencing in breast cancer. Breast Cancer Res Treat.

[112] Li S, Han Z, Zhao N, Zhu B, Zhang Q, Yang X (2018). Inhibition of DNMT suppresses the stemness of colorectal cancer cells through down-regulating wnt signaling pathway. Cell Signal.

[113] Yang Y, Xing Y, Liang C, Hu L, Xu F, Chen Y (2015). Crucial microRNAs and genes of human primary breast cancer explored by microRNA-mRNA integrated analysis. Tumour Biol.

[114] Kim JW, Yang JH, Kim EJ (2020). SIRT1 and AROS suppress doxorubicin-induced apoptosis via inhibition of GSK3beta activity in neuroblastoma cells. Anim Cells Syst (Seoul).

[115] Xin Y, Jin Y, Ge J, Huang Z, Han L, Li C (2021). Involvement of SIRT3-GSK3beta deacetylation pathway in the effects of maternal diabetes on oocyte meiosis. Cell Prolif.

[116] Schlensog M, Magnus L, Heide T, Eschenbruch J, Steib F, Tator M (2018). Epigenetic loss of putative tumor suppressor SFRP3 correlates with poor prognosis of lung adenocarcinoma patients. Epigenetics.

[117] Ai L, Tao Q, Zhong S, Fields CR, Kim WJ, Lee MW (2006). Inactivation of wnt inhibitory factor-1 (WIF1) expression by epigenetic silencing is a common event in breast cancer. Carcinogenesis.

[118] Paluszczak J, Sarbak J, Kostrzewska-Poczekaj M, Kiwerska K, Jarmuż-Szymczak M, Grenman R (2015). The negative regulators of wnt pathway-DACH1, DKK1, and WIF1 are methylated in oral and oropharyngeal cancer and WIF1 methylation predicts shorter survival. Tumour Biol.

[119] Hu H, Li B, Zhou C, Ying X, Chen M, Huang T (2018). Diagnostic value of WIF1 methylation for colorectal cancer: a meta-analysis. Oncotarget.

[120] Zhang B, Ji J, Hu M, Zhou X, Nie Q, Xu H (2023). WIF1 promoter hypermethylation induce endometrial carcinogenesis through the Wnt/beta-catenin signaling pathway. Am J Reprod Immunol.

[121] Liu T, Li Z, Tian F (2021). Quercetin inhibited the proliferation and invasion of hepatoblastoma cells through facilitating SIRT6-medicated FZD4 silence. Hum Exp Toxicol.

[122] Paschidis K, Zougros A, Chatziandreou I, Tsikalakis S, Korkolopoulou P, Kavantzas N (2022). Methylation analysis of APC, AXIN2, DACT1, RASSF1A and MGMT gene promoters in non-small cell lung cancer. Pathol Res Pract.

[123] Richiardi L, Fiano V, Vizzini L, De Marco L, Delsedime L, Akre O (2009). Promoter methylation in APC, RUNX3, and GSTP1 and mortality in prostate cancer patients. J Clin Oncol.

[124] Koinuma K, Yamashita Y, Liu W, Hatanaka H, Kurashina K, Wada T (2006). Epigenetic silencing of AXIN2 in colorectal carcinoma with microsatellite instability. Oncogene.

[125] Li J, Xie G, Tian Y, Li W, Wu Y, Chen F (2022). RNA m(6)a methylation regulates dissemination of cancer cells by modulating expression and membrane localization of β-catenin. Mol Ther.

[126] Schirosi L, Mazzotta A, Opinto G, Pinto R, Graziano G, Tommasi S (2016). β-catenin interaction with NHERF1 and RASSF1A methylation in metastatic colorectal cancer patients. Oncotarget.

[127] Levy L, Wei Y, Labalette C, Wu Y, Renard CA, Buendia MA (2004). Acetylation of beta-catenin by p300 regulates beta-catenin-Tcf4 interaction. Mol Cell Biol.

[128] Wolf D, Rodova M, Miska EA, Calvet JP, Kouzarides T (2002). Acetylation of beta-catenin by CREB-binding protein (CBP). J Biol Chem.

[129] Ge X, Jin Q, Zhang F, Yan T, Zhai Q (2009). PCAF acetylates beta-catenin and improves its stability. Mol Biol Cell.

[130] Kim HY, Choi S, Yoon JH, Lim HJ, Lee H, Choi J (2016). Small molecule inhibitors of the Dishevelled-CXXC5 interaction are new drug candidates for bone anabolic osteoporosis therapy. EMBO Mol Med.

[131] Wu Z, Wei D, Gao W, Xu Y, Hu Z, Ma Z (2015). TPO-Induced metabolic reprogramming drives Liver Metastasis of Colorectal Cancer CD110 + tumor-initiating cells. Cell Stem Cell.

[132] Elfert S, Weise A, Bruser K, Biniossek ML, Jagle S, Senghaas N (2013). Acetylation of human TCF4 (TCF7L2) proteins attenuates inhibition by the HBP1 repressor and induces a conformational change in the TCF4::DNA complex. PLoS ONE.

[133] Song J, Du Z, Ravasz M, Dong B, Wang Z, Ewing RM (2015). A Protein Interaction between β-Catenin and Dnmt1 regulates wnt signaling and DNA methylation in Colorectal Cancer cells. Mol Cancer Res.

[134] Wang Q, Liang N, Yang T, Li Y, Li J, Huang Q (2021). DNMT1-mediated methylation of BEX1 regulates stemness and tumorigenicity in liver cancer. J Hepatol.

[135] Georgescu MM, Gagea M, Cote G (2016). NHERF1/EBP50 suppresses Wnt-β-Catenin pathway-driven intestinal neoplasia. Neoplasia.

[136] Gao C, Xiao G, Hu J (2014). Regulation of Wnt/beta-catenin signaling by posttranslational modifications. Cell Biosci.

[137] Jin H, Luo S, Wang Y, Liu C, Piao Z, Xu M (2017). miR-135b stimulates Osteosarcoma Recurrence and Lung Metastasis via Notch and Wnt/β-Catenin signaling. Mol Ther Nucleic Acids.

[138] Yao X, Mao Y, Wu D, Zhu Y, Lu J, Huang Y (2021). Exosomal circ_0030167 derived from BM-MSCs inhibits the invasion, migration, proliferation and stemness of pancreatic cancer cells by sponging mir-338-5p and targeting the Wif1/Wnt8/β-catenin axis. Cancer Lett.

[139] Lv C, Li F, Li X, Tian Y, Zhang Y, Sheng X (2017). MiR-31 promotes mammary stem cell expansion and breast tumorigenesis by suppressing wnt signaling antagonists. Nat Commun.

[140] Chen D, Li SG, Chen JY, Xiao M (2018). MiR-183 maintains canonical wnt signaling activity and regulates growth and apoptosis in bladder cancer via targeting AXIN2. Eur Rev Med Pharmacol Sci.

[141] Cai J, Guan H, Fang L, Yang Y, Zhu X, Yuan J (2013). MicroRNA-374a activates Wnt/β-catenin signaling to promote breast cancer metastasis. J Clin Invest.

[142] Zhang Y, Wei W, Cheng N, Wang K, Li B, Jiang X (2012). Hepatitis C virus-induced up-regulation of microRNA-155 promotes hepatocarcinogenesis by activating wnt signaling. Hepatology.

[143] Zhang Y, Guo L, Li Y, Feng GH, Teng F, Li W (2018). MicroRNA-494 promotes cancer progression and targets adenomatous polyposis coli in colorectal cancer. Mol Cancer.

[144] Yang XZ, Cheng TT, He QJ, Lei ZY, Chi J, Tang Z (2018). LINC01133 as ceRNA inhibits gastric cancer progression by sponging miR-106a-3p to regulate APC expression and the Wnt/β-catenin pathway. Mol Cancer.

[145] Lu Y, Zhao X, Liu Q, Li C, Graves-Deal R, Cao Z (2017). lncRNA MIR100HG-derived miR-100 and miR-125b mediate cetuximab resistance via Wnt/β-catenin signaling. Nat Med.

[146] Cai J, Fang L, Huang Y, Li R, Xu X, Hu Z (2017). Simultaneous overactivation of Wnt/beta-catenin and TGFbeta signalling by mir-128-3p confers chemoresistance-associated metastasis in NSCLC. Nat Commun.

[147] Wang W, He Y, Rui J, Xu MQ (2019). miR-410 acts as an oncogene in colorectal cancer cells by targeting dickkopf-related protein 1 via the Wnt/β-catenin signaling pathway. Oncol Lett.

[148] Cai J, Fang L, Huang Y, Li R, Xu X, Hu Z (2017). Simultaneous overactivation of Wnt/β-catenin and TGFβ signalling by mir-128-3p confers chemoresistance-associated metastasis in NSCLC. Nat Commun.

[149] Cao MQ, You AB, Zhu XD, Zhang W, Zhang YY, Zhang SZ (2018). Mir-182-5p promotes hepatocellular carcinoma progression by repressing FOXO3a. J Hematol Oncol.

[150] Sun X, Dongol S, Qiu C, Xu Y, Sun C, Zhang Z (2018). miR-652 promotes Tumor Proliferation and Metastasis by Targeting RORA in Endometrial Cancer. Mol Cancer Res.

[151] Wang H, Yan B, Zhang P, Liu S, Li Q, Yang J (2020). MiR-496 promotes migration and epithelial-mesenchymal transition by targeting RASSF6 in colorectal cancer. J Cell Physiol.

[152] Ghafouri-Fard S, Safarzadeh A, Hussen BM, Taheri M, Mokhtari M (2023). Contribution of CRNDE lncRNA in the development of cancer and the underlying mechanisms. Pathol Res Pract.

[153] Zhu L, Yang N, Du G, Li C, Liu G, Liu S (2019). LncRNA CRNDE promotes the epithelial-mesenchymal transition of hepatocellular carcinoma cells via enhancing the Wnt/β-catenin signaling pathway. J Cell Biochem.

[154] Kang Y, Zhang S, Cao W, Wan D, Sun L. Knockdown of LncRNA CRNDE suppresses proliferation and P-glycoprotein-mediated multidrug resistance in acute myelocytic leukemia through the Wnt/β-catenin pathway. Biosci Rep. 2020;40(6). 10.1042/BSR20193450 PMC7273914 32426817

[155] Xu D, Yang F, Yuan JH, Zhang L, Bi HS, Zhou CC (2013). Long noncoding RNAs associated with liver regeneration 1 accelerates hepatocyte proliferation during liver regeneration by activating Wnt/β-catenin signaling. Hepatology.

[156] Ma Y, Yang Y, Wang F, Moyer MP, Wei Q, Zhang P (2016). Long non-coding RNA CCAL regulates colorectal cancer progression by activating Wnt/β-catenin signalling pathway via suppression of activator protein 2α. Gut.

[157] Zhang M, Weng W, Zhang Q, Wu Y, Ni S, Tan C (2018). The lncRNA NEAT1 activates Wnt/β-catenin signaling and promotes colorectal cancer progression via interacting with DDX5. J Hematol Oncol.

[158] Zhu P, Wang Y, Huang G, Ye B, Liu B, Wu J (2016). lnc-β-Catm elicits EZH2-dependent β-catenin stabilization and sustains liver CSC self-renewal. Nat Struct Mol Biol.

[159] Han P, Li JW, Zhang BM, Lv JC, Li YM, Gu XY (2017). The lncRNA CRNDE promotes colorectal cancer cell proliferation and chemoresistance via miR-181a-5p-mediated regulation of Wnt/β-catenin signaling. Mol Cancer.

[160] Luo Y, Huang S, Wei J, Zhou H, Wang W, Yang J (2022). Long noncoding RNA LINC01606 protects colon cancer cells from ferroptotic cell death and promotes stemness by SCD1-Wnt/β-catenin-TFE3 feedback loop signalling. Clin Transl Med.

[161] Liu A, Zhu J, Wu G, Cao L, Tan Z, Zhang S (2017). Antagonizing mir-455-3p inhibits chemoresistance and aggressiveness in esophageal squamous cell carcinoma. Mol Cancer.

[162] Chai S, Ng KY, Tong M, Lau EY, Lee TK, Chan KW (2016). Octamer 4/microRNA-1246 signaling axis drives Wnt/beta-catenin activation in liver cancer stem cells. Hepatology.

[163] Chai S, Ng KY, Tong M, Lau EY, Lee TK, Chan KW (2016). Octamer 4/microRNA-1246 signaling axis drives Wnt/β-catenin activation in liver cancer stem cells. Hepatology.

[164] Han M, Wang S, Fritah S, Wang X, Zhou W, Yang N (2020). Interfering with long non-coding RNA MIR22HG processing inhibits glioblastoma progression through suppression of Wnt/β-catenin signalling. Brain.

[165] Ning MY, Cheng ZL, Zhao J (2020). MicroRNA-448 targets SATB1 to reverse the cisplatin resistance in lung cancer via mediating Wnt/β-catenin signalling pathway. J Biochem.

[166] Belur Nagaraj A, Knarr M, Sekhar S, Connor RS, Joseph P, Kovalenko O (2021). The miR-181a-SFRP4 Axis regulates wnt activation to Drive Stemness and Platinum Resistance in Ovarian Cancer. Cancer Res.

[167] Qian C, Wang B, Zou Y, Zhang Y, Hu X, Sun W (2019). MicroRNA 145 enhances chemosensitivity of glioblastoma stem cells to demethoxycurcumin. Cancer Manag Res.

[168] Ren ZF, Du MF, Fu H, Liu J, Xia FY, Du HN (2020). MiR-200c promotes proliferation of papillary thyroid cancer cells via Wnt/β-catenin signaling pathway. Eur Rev Med Pharmacol Sci.

[169] Sun T, Yin YF, Jin HG, Liu HR, Tian WC (2022). Exosomal microRNA-19b targets FBXW7 to promote colorectal cancer stem cell stemness and induce resistance to radiotherapy. Kaohsiung J Med Sci.

[170] Zhang LY, Chen Y, Jia J, Zhu X, He Y, Wu LM (2019). MiR-27a promotes EMT in ovarian cancer through active Wnt/?-catenin signalling by targeting FOXO1. Cancer Biomark.

[171] Pan J, Fang S, Tian H, Zhou C, Zhao X, Tian H (2020). lncRNA JPX/miR-33a-5p/Twist1 axis regulates tumorigenesis and metastasis of lung cancer by activating Wnt/β-catenin signaling. Mol Cancer.

[172] Zhou S, Eid K, Glowacki J (2004). Cooperation between TGF-beta and wnt pathways during chondrocyte and adipocyte differentiation of human marrow stromal cells. J Bone Min Res.

[173] Letamendia A, Labbé E, Attisano L (2001). Transcriptional regulation by smads: crosstalk between the TGF-beta and wnt pathways. J Bone Joint Surg Am.

[174] Li X, Placencio V, Iturregui JM, Uwamariya C, Sharif-Afshar AR, Koyama T (2008). Prostate tumor progression is mediated by a paracrine TGF-beta/Wnt3a signaling axis. Oncogene.

[175] Ntziachristos P, Lim JS, Sage J, Aifantis I (2014). From fly wings to targeted cancer therapies: a centennial for notch signaling. Cancer Cell.

[176] Estrach S, Ambler CA, Lo Celso C, Hozumi K, Watt FM (2006). Jagged 1 is a beta-catenin target gene required for ectopic hair follicle formation in adult epidermis. Development.

[177] Mangolini M, Götte F, Moore A, Ammon T, Oelsner M, Lutzny-Geier G (2018). Notch2 controls non-autonomous wnt-signalling in chronic lymphocytic leukaemia. Nat Commun.

[178] Sileo P, Simonin C, Melnyk P, Chartier-Harlin MC, Cotelle P. Crosstalk between the hippo pathway and the Wnt pathway in Huntington’s disease and other neurodegenerative disorders. Cells. 2022;11(22). 10.3390/cells11223631 PMC9688160 36429058

[179] Li N, Lu N, Xie C (2019). The Hippo and wnt signalling pathways: crosstalk during neoplastic progression in gastrointestinal tissue. FEBS J.

[180] Azzolin L, Panciera T, Soligo S, Enzo E, Bicciato S, Dupont S (2014). YAP/TAZ incorporation in the β-catenin destruction complex orchestrates the wnt response. Cell.

[181] Varelas X, Miller BW, Sopko R, Song S, Gregorieff A, Fellouse FA (2010). The Hippo pathway regulates Wnt/beta-catenin signaling. Dev Cell.

[182] He J, Sheng T, Stelter AA, Li C, Zhang X, Sinha M (2006). Suppressing wnt signaling by the hedgehog pathway through sFRP-1. J Biol Chem.

[183] Rockel JS, Yu C, Whetstone H, Craft AM, Reilly K, Ma H (2016). Hedgehog inhibits β-catenin activity in synovial joint development and osteoarthritis. J Clin Invest.

[184] Thyssen G, Li TH, Lehmann L, Zhuo M, Sharma M, Sun Z (2006). LZTS2 is a novel beta-catenin-interacting protein and regulates the nuclear export of beta-catenin. Mol Cell Biol.

[185] Ma B, Hottiger MO (2016). Crosstalk between Wnt/beta-Catenin and NF-kappaB signaling pathway during inflammation. Front Immunol.

[186] Cho HH, Song JS, Yu JM, Yu SS, Choi SJ, Kim DH (2008). Differential effect of NF-kappaB activity on beta-catenin/Tcf pathway in various cancer cells. FEBS Lett.

[187] Chang J, Liu F, Lee M, Wu B, Ting K, Zara JN (2013). NF-kappaB inhibits osteogenic differentiation of mesenchymal stem cells by promoting beta-catenin degradation. Proc Natl Acad Sci U S A.

[188] Lamberti C, Lin KM, Yamamoto Y, Verma U, Verma IM, Byers S (2001). Regulation of beta-catenin function by the IkappaB kinases. J Biol Chem.

[189] Albanese C, Wu K, D’Amico M, Jarrett C, Joyce D, Hughes J (2003). IKKalpha regulates mitogenic signaling through transcriptional induction of cyclin D1 via Tcf. Mol Biol Cell.

[190] Carayol N, Wang CY (2006). IKKalpha stabilizes cytosolic beta-catenin by inhibiting both canonical and non-canonical degradation pathways. Cell Signal.

[191] Jeong WJ, Yoon J, Park JC, Lee SH, Lee SH, Kaduwal S (2012). Ras stabilization through aberrant activation of Wnt/β-catenin signaling promotes intestinal tumorigenesis. Sci Signal.

[192] Parsons MJ, Tammela T, Dow LE (2021). WNT as a driver and dependency in Cancer. Cancer Discov.

[193] Basu S, Cheriyamundath S, Ben-Ze’ev A. Cell-cell adhesion: linking Wnt/β-catenin signaling with partial EMT and stemness traits in tumorigenesis. F1000Research. 2018;7. 10.12688/f1000research.15782.1 PMC6144947 30271576

[194] Yook JI, Li XY, Ota I, Fearon ER, Weiss SJ (2005). Wnt-dependent regulation of the E-cadherin repressor snail. J Biol Chem.

[195] Martin-Orozco E, Sanchez-Fernandez A, Ortiz-Parra I, Ayala-San Nicolas M (2019). WNT signaling in tumors: the way to evade drugs and immunity. Front Immunol.

[196] Comprehensive molecular characterization (2012). Of human colon and rectal cancer. Nature.

[197] Kim S, Jeong S (2019). Mutation hotspots in the β-Catenin gene: lessons from the Human Cancer Genome databases. Mol Cells.

[198] Bruun J, Kolberg M, Nesland JM, Svindland A, Nesbakken A, Lothe RA (2014). Prognostic significance of β-Catenin, E-Cadherin, and SOX9 in Colorectal Cancer: results from a large Population-Representative Series. Front Oncol.

[199] Mazzoni SM, Fearon ER (2014). AXIN1 and AXIN2 variants in gastrointestinal cancers. Cancer Lett.

[200] Galiatsatos P, Foulkes WD (2006). Familial adenomatous polyposis. Am J Gastroenterol.

[201] Su LK, Kinzler KW, Vogelstein B, Preisinger AC, Moser AR, Luongo C (1992). Multiple intestinal neoplasia caused by a mutation in the murine homolog of the APC gene. Science.

[202] Barker N, van Es JH, Kuipers J, Kujala P, van den Born M, Cozijnsen M (2007). Identification of stem cells in small intestine and colon by marker gene Lgr5. Nature.

[203] Yanai H, Atsumi N, Tanaka T, Nakamura N, Komai Y, Omachi T (2017). Intestinal cancer stem cells marked by Bmi1 or Lgr5 expression contribute to tumor propagation via clonal expansion. Sci Rep.

[204] Powell AE, Vlacich G, Zhao ZY, McKinley ET, Washington MK, Manning HC (2014). Inducible loss of one apc allele in Lrig1-expressing progenitor cells results in multiple distal colonic tumors with features of familial adenomatous polyposis. Am J Physiol Gastrointest Liver Physiol.

[205] Maynard MA, Ferretti R, Hilgendorf KI, Perret C, Whyte P, Lees JA (2014). Bmi1 is required for tumorigenesis in a mouse model of intestinal cancer. Oncogene.

[206] de Sousa e Melo F, Kurtova AV, Harnoss JM, Kljavin N, Hoeck JD, Hung J (2017). A distinct role for Lgr5(+) stem cells in primary and metastatic colon cancer. Nature.

[207] Nam JS, Turcotte TJ, Smith PF, Choi S, Yoon JK (2006). Mouse cristin/R-spondin family proteins are novel ligands for the frizzled 8 and LRP6 receptors and activate beta-catenin-dependent gene expression. J Biol Chem.

[208] Srivastava A, Rikhari D, Srivastava S (2024). RSPO2 as wnt signaling enabler: important roles in cancer development and therapeutic opportunities. Genes Dis.

[209] Hashimoto T, Ogawa R, Yoshida H, Taniguchi H, Kojima M, Saito Y (2019). EIF3E-RSPO2 and PIEZO1-RSPO2 fusions in colorectal traditional serrated adenoma. Histopathology.

[210] He K, Gan WJ (2023). Wnt/β-Catenin signaling pathway in the Development and Progression of Colorectal Cancer. Cancer Manag Res.

[211] Lee MA, Park JH, Rhyu SY, Oh ST, Kang WK, Kim HN (2014). Wnt3a expression is associated with MMP-9 expression in primary tumor and metastatic site in recurrent or stage IV colorectal cancer. BMC Cancer.

[212] Patel S, Alam A, Pant R, Chattopadhyay S (2019). Wnt signaling and its significance within the Tumor Microenvironment: Novel Therapeutic insights. Front Immunol.

[213] Aizawa T, Karasawa H, Funayama R, Shirota M, Suzuki T, Maeda S (2019). Cancer-associated fibroblasts secrete Wnt2 to promote cancer progression in colorectal cancer. Cancer Med.

[214] Fischer MM, Yeung VP, Cattaruzza F, Hussein R, Yen WC, Murriel C (2017). RSPO3 antagonism inhibits growth and tumorigenicity in colorectal tumors harboring common wnt pathway mutations. Sci Rep.

[215] Toh MR, Wong EYT, Wong SH, Ng AWT, Loo LH, Chow PK (2023). Global Epidemiology and Genetics of Hepatocellular Carcinoma. Gastroenterology.

[216] Hsu HC, Jeng YM, Mao TL, Chu JS, Lai PL, Peng SY (2000). Beta-catenin mutations are associated with a subset of low-stage hepatocellular carcinoma negative for hepatitis B virus and with favorable prognosis. Am J Pathol.

[217] Huang H, Fujii H, Sankila A, Mahler-Araujo BM, Matsuda M, Cathomas G (1999). Beta-catenin mutations are frequent in human hepatocellular carcinomas associated with hepatitis C virus infection. Am J Pathol.

[218] Xu C, Xu Z, Zhang Y, Evert M, Calvisi DF, Chen X. β-Catenin signaling in hepatocellular carcinoma. J Clin Invest. 2022;132(4). 10.1172/JCI154515 PMC8843739 35166233

[219] Audard V, Grimber G, Elie C, Radenen B, Audebourg A, Letourneur F (2007). Cholestasis is a marker for hepatocellular carcinomas displaying beta-catenin mutations. J Pathol.

[220] Tao J, Xu E, Zhao Y, Singh S, Li X, Couchy G (2016). Modeling a human hepatocellular carcinoma subset in mice through coexpression of met and point-mutant β-catenin. Hepatology.

[221] Stauffer JK, Scarzello AJ, Andersen JB, De Kluyver RL, Back TC, Weiss JM (2011). Coactivation of AKT and β-catenin in mice rapidly induces formation of lipogenic liver tumors. Cancer Res.

[222] Charawi S, Just PA, Savall M, Abitbol S, Traore M, Metzger N (2019). LKB1 signaling is activated in CTNNB1-mutated HCC and positively regulates β-catenin-dependent CTNNB1-mutated HCC. J Pathol.

[223] Tao J, Krutsenko Y, Moghe A, Singh S, Poddar M, Bell A (2021). Nuclear factor erythroid 2-related factor 2 and β-Catenin coactivation in Hepatocellular Cancer: Biological and therapeutic implications. Hepatology.

[224] Zhan N, Michael AA, Wu K, Zeng G, Bell A, Tao J (2018). The effect of selective c-MET inhibitor on Hepatocellular Carcinoma in the MET-Active, β-Catenin-mutated mouse model. Gene Expr.

[225] Abitbol S, Dahmani R, Coulouarn C, Ragazzon B, Mlecnik B, Senni N (2018). AXIN deficiency in human and mouse hepatocytes induces hepatocellular carcinoma in the absence of β-catenin activation. J Hepatol.

[226] Yang X, Xiang X, Xu G, Zhou S, An T, Huang Z (2023). Silencing of peroxiredoxin 2 suppresses proliferation and Wnt/β-catenin pathway, and induces senescence in hepatocellular carcinoma. Oncol Res.

[227] Di LJ, Byun JS, Wong MM, Wakano C, Taylor T, Bilke S (2013). Genome-wide profiles of CtBP link metabolism with genome stability and epithelial reprogramming in breast cancer. Nat Commun.

[228] Brunquell C, Biliran H, Jennings S, Ireland SK, Chen R, Ruoslahti E (2012). TLE1 is an anoikis regulator and is downregulated by Bit1 in breast cancer cells. Mol cancer Research: MCR.

[229] Geyer FC, Lacroix-Triki M, Savage K, Arnedos M, Lambros MB, MacKay A (2011). β-Catenin pathway activation in breast cancer is associated with triple-negative phenotype but not with CTNNB1 mutation. Mod Pathology: Official J United States Can Acad Pathol Inc.

[230] Hashizume R, Koizumi H, Ihara A, Ohta T, Uchikoshi T (1996). Expression of beta-catenin in normal breast tissue and breast carcinoma: a comparative study with epithelial cadherin and alpha-catenin. Histopathology.

[231] Xu X, Zhang M, Xu F, Jiang S (2020). Wnt signaling in breast cancer: biological mechanisms, challenges and opportunities. Mol Cancer.

[232] Cui J, Li P, Liu X, Hu H, Wei W (2015). Abnormal expression of the Notch and Wnt/β-catenin signaling pathways in stem-like ALDH(hi)CD44(+) cells correlates highly with Ki-67 expression in breast cancer. Oncol Lett.

[233] Jang GB, Hong IS, Kim RJ, Lee SY, Park SJ, Lee ES (2015). Wnt/β-Catenin small-molecule inhibitor CWP232228 preferentially inhibits the growth of breast Cancer stem-like cells. Cancer Res.

[234] Francis JC, Thomsen MK, Taketo MM, Swain A (2013). β-catenin is required for prostate development and cooperates with Pten loss to drive invasive carcinoma. PLoS Genet.

[235] Kypta RM, Waxman J (2012). Wnt/β-catenin signalling in prostate cancer. Nat Reviews Urol.

[236] Yokoyama NN, Shao S, Hoang BH, Mercola D, Zi X (2014). Wnt signaling in castration-resistant prostate cancer: implications for therapy. Am J Clin Experimental Urol.

[237] Robinson D, Van Allen EM, Wu YM, Schultz N, Lonigro RJ, Mosquera JM (2015). Integrative clinical genomics of advanced prostate cancer. Cell.

[238] Valkenburg KC, Hostetter G, Williams BO (2015). Concurrent hepsin overexpression and adenomatous polyposis coli deletion causes invasive prostate carcinoma in mice. Prostate.

[239] Sun Y, Campisi J, Higano C, Beer TM, Porter P, Coleman I (2012). Treatment-induced damage to the tumor microenvironment promotes prostate cancer therapy resistance through WNT16B. Nat Med.

[240] Xue G, Romano E, Massi D, Mandalà M (2016). Wnt/β-catenin signaling in melanoma: preclinical rationale and novel therapeutic insights. Cancer Treat Rev.

[241] Hartman ML, Talar B, Gajos-Michniewicz A, Czyz M (2015). MCL-1, BCL-XL and MITF are diversely employed in adaptive response of Melanoma Cells to changes in Microenvironment. PLoS ONE.

[242] Wei CY, Zhu MX, Yang YW, Zhang PF, Yang X, Peng R (2019). Downregulation of RNF128 activates Wnt/β-catenin signaling to induce cellular EMT and stemness via CD44 and CTTN ubiquitination in melanoma. J Hematol Oncol.

[243] Chien AJ, Moore EC, Lonsdorf AS, Kulikauskas RM, Rothberg BG, Berger AJ (2009). Activated Wnt/β-catenin signaling in melanoma is associated with decreased proliferation in patient tumors and a murine melanoma model. Proc Natl Acad Sci USA.

[244] Bachmann IM, Straume O, Puntervoll HE, Kalvenes MB, Akslen LA (2005). Importance of P-cadherin, β-catenin, and Wnt5a/Frizzled for progression of melanocytic tumors and prognosis in cutaneous melanoma. Clin Cancer Res.

[245] Arozarena I, Bischof H, Gilby D, Belloni B, Dummer R, Wellbrock C (2011). In melanoma, beta-catenin is a suppressor of invasion. Oncogene.

[246] Kageshita T, Hamby CV, Ishihara T, Matsumoto K, Saida T, Ono T (2001). Loss of β-catenin expression associated with disease progression in malignant melanoma. Br J Dermatol.

[247] Da Forno PD, Pringle JH, Hutchinson P, Osborn J, Huang Q, Potter L (2008). WNT5A expression increases during melanoma progression and correlates with outcome. Clin cancer Research: Official J Am Association Cancer Res.

[248] Weeraratna AT, Jiang Y, Hostetter G, Rosenblatt K, Duray P, Bittner M (2002). Wnt5a signaling directly affects cell motility and invasion of metastatic melanoma. Cancer Cell.

[249] Kruck S, Eyrich C, Scharpf M, Sievert KD, Fend F, Stenzl A (2013). Impact of an altered Wnt1/β-catenin expression on clinicopathology and prognosis in clear cell renal cell carcinoma. Int J Mol Sci.

[250] Hsu RJ, Ho JY, Cha TL, Yu DS, Wu CL, Huang WP (2012). WNT10A plays an oncogenic role in renal cell carcinoma by activating WNT/β-catenin pathway. PLoS ONE.

[251] Tamimi Y, Ekuere U, Laughton N, Grundy P (2008). WNT5A is regulated by PAX2 and may be involved in blastemal predominant Wilms tumorigenesis. Neoplasia (New York NY).

[252] Kondratov AG, Kvasha SM, Stoliar LA, Romanenko AM, Zgonnyk YM, Gordiyuk VV (2012). Alterations of the WNT7A gene in clear cell renal cell carcinomas. PLoS ONE.

[253] Janssens N, Andries L, Janicot M, Perera T, Bakker A (2004). Alteration of frizzled expression in renal cell carcinoma. Tumour Biology: J Int Soc Oncodevelopmental Biology Med.

[254] Song H, Nan Y, Wang X, Zhang G, Zong S, Kong X (2017). MicroRNA–613 inhibits proliferation and invasion of renal cell carcinoma cells through targeting FZD7. Mol Med Rep.

[255] Bilim V, Kawasaki T, Katagiri A, Wakatsuki S, Takahashi K, Tomita Y (2000). Altered expression of beta-catenin in renal cell cancer and transitional cell cancer with the absence of beta-catenin gene mutations. Clin cancer Research: Official J Am Association Cancer Res.

[256] Xu Q, Krause M, Samoylenko A, Vainio S. Wnt signaling in renal cell carcinoma. Cancers. 2016;8(6). 10.3390/cancers8060057 PMC4931622 27322325

[257] Saini S, Majid S, Dahiya R (2011). The complex roles of wnt antagonists in RCC. Nat Reviews Urol.

[258] Guo CC, Zhang XL, Yang B, Geng J, Peng B, Zheng JH (2014). Decreased expression of Dkk1 and Dkk3 in human clear cell renal cell carcinoma. Mol Med Rep.

[259] Ueno K, Hirata H, Majid S, Chen Y, Zaman MS, Tabatabai ZL (2011). Wnt antagonist DICKKOPF-3 (Dkk-3) induces apoptosis in human renal cell carcinoma. Mol Carcinog.

[260] Lorzadeh S, Kohan L, Ghavami S, Azarpira N (2021). Autophagy and the wnt signaling pathway: a focus on Wnt/β-catenin signaling. Biochim et Biophys acta Mol cell Res.

[261] Barzegar Behrooz A, Talaie Z, Jusheghani F, Łos MJ, Klonisch T, Ghavami S. Wnt and PI3K/Akt/mTOR survival pathways as therapeutic targets in Glioblastoma. Int J Mol Sci. 2022;23(3). 10.3390/ijms23031353 PMC8836096 35163279

[262] Nager M, Bhardwaj D, Cantí C, Medina L, Nogués P, Herreros J (2012). β-Catenin signalling in Glioblastoma Multiforme and Glioma-initiating cells. Chemother Res Pract.

[263] Iwadate Y (2016). Epithelial-mesenchymal transition in glioblastoma progression. Oncol Lett.

[264] Kahlert UD, Nikkhah G, Maciaczyk J (2013). Epithelial-to-mesenchymal(-like) transition as a relevant molecular event in malignant gliomas. Cancer Lett.

[265] Tang N, Song WX, Luo J, Haydon RC, He TC (2008). Osteosarcoma development and stem cell differentiation. Clin Orthop Relat Res.

[266] Mohseny AB, Szuhai K, Romeo S, Buddingh EP, Briaire-de Bruijn I, de Jong D (2009). Osteosarcoma originates from mesenchymal stem cells in consequence of aneuploidization and genomic loss of Cdkn2. J Pathol.

[267] Brune JC, Tormin A, Johansson MC, Rissler P, Brosjö O, Löfvenberg R (2011). Mesenchymal stromal cells from primary osteosarcoma are non-malignant and strikingly similar to their bone marrow counterparts. Int J Cancer.

[268] Chen C, Zhao M, Tian A, Zhang X, Yao Z, Ma X (2015). Aberrant activation of Wnt/β-catenin signaling drives proliferation of bone sarcoma cells. Oncotarget.

[269] Iwaya K, Ogawa H, Kuroda M, Izumi M, Ishida T, Mukai K (2003). Cytoplasmic and/or nuclear staining of beta-catenin is associated with lung metastasis. Clin Exp Metastasis.

[270] Lu Y, Guan GF, Chen J, Hu B, Sun C, Ma Q (2015). Aberrant CXCR4 and β-catenin expression in osteosarcoma correlates with patient survival. Oncol Lett.

[271] Du X, Yang J, Yang D, Tian W, Zhu Z (2014). The genetic basis for inactivation of wnt pathway in human osteosarcoma. BMC Cancer.

[272] Danieau G, Morice S, Rédini F, Verrecchia F, Royer BB. New insights about the Wnt/β-Catenin signaling pathway in primary bone tumors and their microenvironment: a promising target to develop therapeutic strategies? Int J Mol Sci. 2019;20(15). 10.3390/ijms20153751 PMC6696068 31370265

[273] Lu Q, Huang H, Wang X, Luo L, Xia H, Zhang L (2023). Echinatin inhibits the growth and metastasis of human osteosarcoma cells through Wnt/β-catenin and p38 signaling pathways. Pharmacol Res.

[274] Guan J, He J, Liao S, Wu Z, Lin X, Liu B (2022). LncRNA UCA1 accelerates osteosarcoma progression via miR-145 and Wnt/β-catenin pathway. Am J Translational Res.

[275] Li C, Shi X, Zhou G, Liu X, Wu S, Zhao J (2013). The canonical wnt-beta-catenin pathway in development and chemotherapy of osteosarcoma. FBL.

[276] Cai Y, Mohseny AB, Karperien M, Hogendoorn PC, Zhou G, Cleton-Jansen AM (2010). Inactive Wnt/beta-catenin pathway in conventional high-grade osteosarcoma. J Pathol.

[277] Zhang X, Hao J (2015). Development of anticancer agents targeting the Wnt/β-catenin signaling. Am J Cancer Res.

[278] Krishnamurthy N, Kurzrock R (2018). Targeting the Wnt/beta-catenin pathway in cancer: update on effectors and inhibitors. Cancer Treat Rev.

[279] Agarwal P, Zhang B, Ho Y, Cook A, Li L, Mikhail FM (2017). Enhanced targeting of CML stem and progenitor cells by inhibition of porcupine acyltransferase in combination with TKI. Blood.

[280] Liu J, Pan S, Hsieh MH, Ng N, Sun F, Wang T (2013). Targeting wnt-driven cancer through the inhibition of Porcupine by LGK974. Proc Natl Acad Sci USA.

[281] Doo DW, Meza-Perez S, Londoño AI, Goldsberry WN, Katre AA, Boone JD (2020). Inhibition of the Wnt/β-catenin pathway enhances antitumor immunity in ovarian cancer. Therapeutic Adv Med Oncol.

[282] Madan B, Ke Z, Harmston N, Ho SY, Frois AO, Alam J (2016). Wnt addiction of genetically defined cancers reversed by PORCN inhibition. Oncogene.

[283] Goldsberry WN, Meza-Perez S, Londoño AI, Katre AA, Mott BT, Roane BM et al. Inhibiting WNT ligand production for improved immune recognition in the ovarian tumor microenvironment. Cancers. 2020;12(3). 10.3390/cancers12030766 PMC7140065 32213921

[284] Yang Q, Qin T, An T, Wu H, Xu G, Xiang J (2023). Novel PORCN inhibitor WHN-88 targets Wnt/beta-catenin pathway and prevents the growth of wnt-driven cancers. Eur J Pharmacol.

[285] Neiheisel A, Kaur M, Ma N, Havard P, Shenoy AK (2022). Wnt pathway modulators in cancer therapeutics: an update on completed and ongoing clinical trials. Int J Cancer.

[286] Phillips C, Bhamra I, Eagle C, Flanagan E, Armer R, Jones CD (2022). The wnt pathway inhibitor RXC004 blocks Tumor Growth and reverses Immune Evasion in wnt ligand-dependent Cancer models. Cancer Res Commun.

[287] Shah K, Panchal S, Patel B (2021). Porcupine inhibitors: Novel and emerging anti-cancer therapeutics targeting the wnt signaling pathway. Pharmacol Res.

[288] Zhong Z, Sepramaniam S, Chew XH, Wood K, Lee MA, Madan B (2019). PORCN inhibition synergizes with PI3K/mTOR inhibition in wnt-addicted cancers. Oncogene.

[289] Cheng D, Liu J, Han D, Zhang G, Gao W, Hsieh MH (2016). Discovery of Pyridinyl Acetamide derivatives as potent, selective, and orally bioavailable porcupine inhibitors. ACS Med Chem Lett.

[290] Larasati Y, Boudou C, Koval A, Katanaev VL (2022). Unlocking the wnt pathway: therapeutic potential of selective targeting FZD(7) in cancer. Drug Discov Today.

[291] Li Y, Li PK, Roberts MJ, Arend RC, Samant RS, Buchsbaum DJ (2014). Multi-targeted therapy of cancer by niclosamide: a new application for an old drug. Cancer Lett.

[292] Choi J, Lee K, Kang M, Lim SK, Tai No K (2018). In silico discovery of quinoxaline derivatives as novel LRP5/6-sclerostin interaction inhibitors. Bioorg Med Chem Lett.

[293] Lu W, Liu CC, Thottassery JV, Bu G, Li Y (2010). Mesd is a universal inhibitor of wnt coreceptors LRP5 and LRP6 and blocks Wnt/beta-catenin signaling in cancer cells. Biochemistry.

[294] Jung YS, Park JI (2020). Wnt signaling in cancer: therapeutic targeting of wnt signaling beyond beta-catenin and the destruction complex. Exp Mol Med.

[295] Xue W, Zhu B, Zhao K, Huang Q, Luo H, Shou Y (2024). Targeting LRP6: a new strategy for cancer therapy. Pharmacol Res.

[296] Zhang X, Zhu J, Yang GY, Wang QJ, Qian L, Chen YM (2007). Dishevelled promotes axon differentiation by regulating atypical protein kinase C. Nat Cell Biol.

[297] Huang S, Chen J, Tian R, Wang J, Xie C, Gao H (2018). Down-regulation of dishevelled-2 inhibits cell proliferation and invasion in hepatoblastoma. Pediatr Blood Cancer.

[298] Kang HE, Seo Y, Yun JS, Song SH, Han D, Cho ES et al. Metformin and niclosamide synergistically suppress Wnt and YAP in APC-mutated colorectal cancer. Cancers (Basel). 2021;13(14). 10.3390/cancers13143437 PMC8308039 34298652

[299] Fujii N, You L, Xu Z, Uematsu K, Shan J, He B (2007). An antagonist of dishevelled protein-protein interaction suppresses beta-catenin-dependent tumor cell growth. Cancer Res.

[300] Grandy D, Shan J, Zhang X, Rao S, Akunuru S, Li H (2009). Discovery and characterization of a small molecule inhibitor of the PDZ domain of dishevelled. J Biol Chem.

[301] Trujano-Camacho S, Cantu-de Leon D, Delgado-Waldo I, Coronel-Hernandez J, Millan-Catalan O, Hernandez-Sotelo D (2021). Inhibition of wnt-beta-catenin signaling by ICRT14 drug depends of Post-transcriptional Regulation by HOTAIR in Human cervical Cancer HeLa cells. Front Oncol.

[302] Ma S, Choi J, Jin X, Kim HY, Yun JH, Lee W (2018). Discovery of a small-molecule inhibitor of Dvl-CXXC5 interaction by computational approaches. J Comput Aided Mol Des.

[303] Lee HJ, Wang NX, Shi DL, Zheng JJ (2009). Sulindac inhibits canonical wnt signaling by blocking the PDZ domain of the protein dishevelled. Angewandte Chemie (International ed English).

[304] Lee E, Salic A, Krüger R, Heinrich R, Kirschner MW (2003). The roles of APC and axin derived from experimental and theoretical analysis of the wnt pathway. PLoS Biol.

[305] Bernkopf DB, Brückner M, Hadjihannas MV, Behrens J (2019). An aggregon in conductin/axin2 regulates Wnt/β-catenin signaling and holds potential for cancer therapy. Nat Commun.

[306] Ji L, Lu B, Zamponi R, Charlat O, Aversa R, Yang Z (2019). USP7 inhibits Wnt/β-catenin signaling through promoting stabilization of Axin. Nat Commun.

[307] Kim MK (2018). Novel insight into the function of tankyrase. Oncol Lett.

[308] Huang SM, Mishina YM, Liu S, Cheung A, Stegmeier F, Michaud GA (2009). Tankyrase inhibition stabilizes axin and antagonizes wnt signalling. Nature.

[309] Lau T, Chan E, Callow M, Waaler J, Boggs J, Blake RA (2013). A novel tankyrase small-molecule inhibitor suppresses APC mutation-driven colorectal tumor growth. Cancer Res.

[310] Mizutani A, Yashiroda Y, Muramatsu Y, Yoshida H, Chikada T, Tsumura T (2018). RK-287107, a potent and specific tankyrase inhibitor, blocks colorectal cancer cell growth in a preclinical model. Cancer Sci.

[311] Waaler J, Machon O, Tumova L, Dinh H, Korinek V, Wilson SR (2012). A novel tankyrase inhibitor decreases canonical wnt signaling in colon carcinoma cells and reduces tumor growth in conditional APC mutant mice. Cancer Res.

[312] Okada-Iwasaki R, Takahashi Y, Watanabe Y, Ishida H, Saito J, Nakai R (2016). The Discovery and characterization of K-756, a Novel Wnt/β-Catenin pathway inhibitor targeting Tankyrase. Mol Cancer Ther.

[313] Busch AM, Johnson KC, Stan RV, Sanglikar A, Ahmed Y, Dmitrovsky E (2013). Evidence for tankyrases as antineoplastic targets in lung cancer. BMC Cancer.

[314] Menon M, Elliott R, Bowers L, Balan N, Rafiq R, Costa-Cabral S (2019). A novel tankyrase inhibitor, MSC2504877, enhances the effects of clinical CDK4/6 inhibitors. Sci Rep.

[315] Quackenbush KS, Bagby S, Tai WM, Messersmith WA, Schreiber A, Greene J (2016). The novel tankyrase inhibitor (AZ1366) enhances irinotecan activity in tumors that exhibit elevated tankyrase and irinotecan resistance. Oncotarget.

[316] Stratford EW, Daffinrud J, Munthe E, Castro R, Waaler J, Krauss S (2014). The tankyrase-specific inhibitor JW74 affects cell cycle progression and induces apoptosis and differentiation in osteosarcoma cell lines. Cancer Med.

[317] Arqués O, Chicote I, Puig I, Tenbaum SP, Argilés G, Dienstmann R (2016). Tankyrase Inhibition Blocks Wnt/β-Catenin pathway and reverts resistance to PI3K and AKT inhibitors in the treatment of Colorectal Cancer. Clin Cancer Res.

[318] Shen C, Nayak A, Neitzel LR, Adams AA, Silver-Isenstadt M, Sawyer LM (2021). The E3 ubiquitin ligase component, Cereblon, is an evolutionarily conserved regulator of wnt signaling. Nat Commun.

[319] Thorne CA, Hanson AJ, Schneider J, Tahinci E, Orton D, Cselenyi CS (2010). Small-molecule inhibition of wnt signaling through activation of casein kinase 1α. Nat Chem Biol.

[320] Fan J, Reid RR, He TC (2022). Pyrvinium doubles against WNT-driven cancer. J Biol Chem.

[321] Li B, Orton D, Neitzel LR, Astudillo L, Shen C, Long J et al. Differential abundance of CK1α provides selectivity for pharmacological CK1α activators to target WNT-dependent tumors. Sci Signal. 2017;10(485). 10.1126/scisignal.aak9916 PMC5555225 28655862

[322] Rodriguez-Blanco J, Li B, Long J, Shen C, Yang F, Orton D (2019). A CK1α activator penetrates the brain and shows efficacy against drug-resistant metastatic medulloblastoma. Clin cancer Research: Official J Am Association Cancer Res.

[323] Lepourcelet M, Chen YN, France DS, Wang H, Crews P, Petersen F (2004). Small-molecule antagonists of the oncogenic Tcf/beta-catenin protein complex. Cancer Cell.

[324] Sukhdeo K, Mani M, Zhang Y, Dutta J, Yasui H, Rooney MD (2007). Targeting the beta-catenin/TCF transcriptional complex in the treatment of multiple myeloma. Proc Natl Acad Sci U S A.

[325] Minke KS, Staib P, Puetter A, Gehrke I, Gandhirajan RK, Schlösser A (2009). Small molecule inhibitors of WNT signaling effectively induce apoptosis in acute myeloid leukemia cells. Eur J Haematol.

[326] Gandhirajan RK, Staib PA, Minke K, Gehrke I, Plickert G, Schlösser A (2010). Small molecule inhibitors of Wnt/beta-catenin/lef-1 signaling induces apoptosis in chronic lymphocytic leukemia cells in vitro and in vivo. Neoplasia.

[327] Wei W, Chua MS, Grepper S, So S (2010). Small molecule antagonists of Tcf4/beta-catenin complex inhibit the growth of HCC cells in vitro and in vivo. Int J Cancer.

[328] Yamashita T, Budhu A, Forgues M, Wang XW (2007). Activation of hepatic stem cell marker EpCAM by wnt-beta-catenin signaling in hepatocellular carcinoma. Cancer Res.

[329] Catrow JL, Zhang Y, Zhang M, Ji H (2015). Discovery of Selective Small-Molecule inhibitors for the β-Catenin/T-Cell factor protein-protein Interaction through the optimization of the Acyl Hydrazone Moiety. J Med Chem.

[330] Wang XH, Sun X, Meng XW, Lv ZW, Du YJ, Zhu Y (2010). beta-catenin siRNA regulation of apoptosis- and angiogenesis-related gene expression in hepatocellular carcinoma cells: potential uses for gene therapy. Oncol Rep.

[331] Xiang Q, Wang C, Zhang Y, Xue X, Song M, Zhang C (2018). Discovery and optimization of 1-(1H-indol-1-yl)ethanone derivatives as CBP/EP300 bromodomain inhibitors for the treatment of castration-resistant prostate cancer. Eur J Med Chem.

[332] Romero FA, Murray J, Lai KW, Tsui V, Albrecht BK, An L (2017). GNE-781, a highly advanced potent and selective bromodomain inhibitor of cyclic Adenosine Monophosphate response element binding protein, binding protein (CBP). J Med Chem.

[333] Evangelisti C, Chiarini F, Cappellini A, Paganelli F, Fini M, Santi S (2020). Targeting Wnt/β-catenin and PI3K/Akt/mTOR pathways in T-cell acute lymphoblastic leukemia. J Cell Physiol.

[334] Cianciosi D, Varela-Lopez A, Forbes-Hernandez TY, Gasparrini M, Afrin S, Reboredo-Rodriguez P (2018). Targeting molecular pathways in cancer stem cells by natural bioactive compounds. Pharmacol Res.

[335] Lenz HJ, Kahn M (2014). Safely targeting cancer stem cells via selective catenin coactivator antagonism. Cancer Sci.

[336] Osawa Y, Oboki K, Imamura J, Kojika E, Hayashi Y, Hishima T (2015). Inhibition of cyclic Adenosine Monophosphate (cAMP)-response element-binding protein (CREB)-binding protein (CBP)/β-Catenin reduces liver fibrosis in mice. EBioMedicine.

[337] Wu G, Cao L, Zhu J, Tan Z, Tang M, Li Z (2019). Loss of RBMS3 confers Platinum Resistance in Epithelial Ovarian Cancer via activation of miR-126-5p/β-catenin/CBP signaling. Clin Cancer Res.

[338] Warner DR, Smith SC, Smolenkova IA, Pisano MM, Greene RM (2016). Inhibition of p300 histone acetyltransferase activity in palate mesenchyme cells attenuates wnt signaling via aberrant E-cadherin expression. Exp Cell Res.

[339] Arensman MD, Telesca D, Lay AR, Kershaw KM, Wu N, Donahue TR (2014). The CREB-binding protein inhibitor ICG-001 suppresses pancreatic cancer growth. Mol Cancer Ther.

[340] Won HS, Lee KM, Oh JE, Nam EM, Lee KE (2016). Inhibition of beta-catenin to overcome endocrine resistance in tamoxifen-resistant breast Cancer Cell line. PLoS ONE.

[341] Zhang Y, Wang X (2020). Targeting the Wnt/β-catenin signaling pathway in cancer. J Hematol Oncol.

[342] Prabhu VV, Lulla AR, Madhukar NS, Ralff MD, Zhao D, Kline CLB (2017). Cancer stem cell-related gene expression as a potential biomarker of response for first-in-class imipridone ONC201 in solid tumors. PLoS ONE.

[343] Wu L, He S, He Y, Wang X, Lu L (2018). IC-2 suppresses proliferation and induces apoptosis of bladder Cancer cells via the Wnt/beta-Catenin pathway. Med Sci Monit.

[344] Urushibara S, Tsubota T, Asai R, Azumi J, Ashida K, Fujiwara Y (2017). WNT/beta-Catenin signaling inhibitor IC-2 suppresses sphere formation and sensitizes colorectal Cancer cells to 5-Fluorouracil. Anticancer Res.

[345] Seto K, Sakabe T, Itaba N, Azumi J, Oka H, Morimoto M (2017). A novel small-molecule WNT inhibitor, IC-2, has the potential to suppress Liver Cancer Stem cells. Anticancer Res.

[346] Chen Y, Rao X, Huang K, Jiang X, Wang H, Teng L (2017). FH535 inhibits proliferation and motility of Colon cancer cells by targeting Wnt/beta-catenin signaling pathway. J Cancer.

[347] Yu F, Yu C, Li F, Zuo Y, Wang Y, Yao L (2021). Wnt/β-catenin signaling in cancers and targeted therapies. Signal Transduct Target Ther.

[348] Flanagan DJ, Vincan E, Phesse TJ (2019). Wnt signaling in Cancer: not a binary ON:OFF switch. Cancer Res.

[349] Le PN, McDermott JD, Jimeno A (2015). Targeting the wnt pathway in human cancers: therapeutic targeting with a focus on OMP-54F28. Pharmacol Ther.

[350] Wang Z, Li Z, Ji H (2021). Direct targeting of β-catenin in the wnt signaling pathway: current progress and perspectives. Med Res Rev.

[351] Jung YS, Park JI (2020). Wnt signaling in cancer: therapeutic targeting of wnt signaling beyond β-catenin and the destruction complex. Exp Mol Med.

[352] van Andel H, Kocemba KA, Spaargaren M, Pals ST (2019). Aberrant wnt signaling in multiple myeloma: molecular mechanisms and targeting options. Leukemia.

[353] Choi RB, Hoggatt AM, Horan DJ, Rogers EZ, Hong JM, Robling AG (2022). Targeting Sclerostin and Dkk1 at optimized proportions of low-dose antibody achieves similar skeletal benefits to higher-dose sclerostin targeting in the mature adult and aged Skeleton. Aging Dis.

[354] Zhang Q, Wang L, Wang S, Cheng H, Xu L, Pei G (2022). Signaling pathways and targeted therapy for myocardial infarction. Signal Transduct Target Ther.

[355] Zhao H, Ming T, Tang S, Ren S, Yang H, Liu M (2022). Wnt signaling in colorectal cancer: pathogenic role and therapeutic target. Mol Cancer.

[356] Hung SW, Zhang R, Tan Z, Chung JPW, Zhang T, Wang CC (2021). Pharmaceuticals targeting signaling pathways of endometriosis as potential new medical treatment: a review. Med Res Rev.

[357] Liu J, Xiao Q, Xiao J, Niu C, Li Y, Zhang X (2022). Wnt/β-catenin signalling: function, biological mechanisms, and therapeutic opportunities. Signal Transduct Target Ther.

[358] Cui C, Zhou X, Zhang W, Qu Y, Ke X (2018). Is β-Catenin a Druggable Target for Cancer Therapy?. Trends Biochem Sci.

[359] Kahn M (2014). Can we safely target the WNT pathway?. Nat Rev Drug Discov.

